# Exploring mosquito virome dynamics within São Paulo Zoo: insights into mosquito-virus-environment interactions

**DOI:** 10.3389/fcimb.2024.1496126

**Published:** 2025-01-10

**Authors:** Lilian de Oliveira Guimarães, Geovani de Oliveira Ribeiro, Roseane da Couto, Endrya do Socorro Foro Ramos, Vanessa dos Santos Morais, Juliana Telles-de-Deus, Vanessa Christe Helfstein, Jesus Maia dos Santos, Xutao Deng, Eric Delwart, Ramendra Pati Pandey, Vera Lucia Fonseca de Camargo-Neves, Antonio Charlys da Costa, Karin Kirchgatter, Élcio Leal

**Affiliations:** ^1^ Instituto Pasteur, São Paulo, SP, Brazil; ^2^ General-Coordination of Public Health Laboratories, Health and Environment Surveillance Secretariat, Ministry of Health, Brasilia, Brazil; ^3^ Department of Cellular Biology, University of Brasilia (UNB), Brasilia, Brazil; ^4^ Institute of Biological Sciences, Federal University of Pará, Belem, Pará, Brazil; ^5^ Instituto de Medicina Tropical, Faculdade de Medicina, Universidade de São Paulo, São Paulo, SP, Brazil; ^6^ Vitalant Research Institute, San Francisco, CA, United States; ^7^ Department Laboratory Medicine, University of California, San Francisco, San Francisco, CA, United States; ^8^ School of Health Sciences and Technology (SoHST), University of Petroleum and Energy Studies (UPES), Dehradun, Uttarakhand, India

**Keywords:** metagenomics, virome, mosquitoes, São Paulo Zoo, vector-borne arbovirus

## Abstract

**Background:**

Mosquito-borne diseases have a significant public health threat worldwide, with arboviruses accounting for a high proportion of infectious diseases and mortality annually. Brazil, in particular, has been suffering outbreaks of diseases transmitted by mosquito viruses, notably those of the *Aedes* genus, such as dengue, Zika, and chikungunya. Against this background, the São Paulo Zoo is an intriguing ecological niche to explore the virome of mosquitoes, potentially shedding light on the dynamics of arbovirus transmission within a confined setting.

**Methods:**

In this study, we conducted a comprehensive metagenomic analysis of mosquitoes collected from diverse habitats within the zoo, focusing on the *Aedes*, *Anopheles*, and *Culex* genera. From 1,039 contigs of viral origin, we identified 229 viral species infecting mosquitoes, with the orders *Picornavirales*, *Nodamuvirales* and *Sobelivirales* being the most prevalent and abundant. The difference in virome composition was primarily driven by mosquito host species rather than specific collection sites or trap height.

**Results:**

Despite environmental disparities, the virome remained remarkably uniform across different areas of the zoo, emphasizing the strong association between mosquito species and their viral communities. Furthermore, we identified a core virome shared among mosquito species, highlighting potential cross-species transmission events and underscoring the need for targeted surveillance and control measures.

**Conclusion:**

These results contribute to our understanding of the interplay between mosquitoes, the environment, and viruses, providing valuable insights for disease intervention strategies in mosquito-borne diseases.

## 1 Introduction

According to WHO, vector-borne diseases contribute to more than 17% of infectious diseases, resulting in 700,000 deaths annually (World Health Organization, 2020). Mosquitoes are primary carriers, transmitting over a third of arthropod-borne viruses (arboviruses) (Almeida et al., 2021). In recent decades, significant outbreaks of diseases transmitted by mosquitoes, such as mosquitoes of the Culicinae subfamily of the genus *Aedes*, have been observed, transmitting diseases such as dengue, Zika, chikungunya, and yellow fever. In Brazil, the Chikungunya and dengue viruses have caused numerous severe epidemics in recent years. In 2023, Brazil had one of the most significant dengue outbreaks recorded, with more than 1.6 million reported cases and more than 1,000 deaths (Brasil, 2023). The following year, 2024, was marked by another explosion in cases, with more than 4 million cases reported until May 2024, resulting in 2500 deaths (Brasil, 2024). In recent years factors like prolonged temperature anomalies, rising population density, and increasing urbanization are driving a concerning rise in arbovirus incidence across Brazil (Barcellos et al., 2024).

Mosquitoes belonging to the family Culicidae (order Diptera, class Insecta, subphylum Hexapoda, and phylum Arthropoda) stand out for their high diversity and geographical distribution, especially in South America, with Brazil being particularly noteworthy (Harbach, 2013). This is due to Brazil’s vast dimensions and its richness in fauna and flora, as well as its diverse biomes (Ribeiro et al., 2020). This environmental diversity facilitates the circulation and interaction of mosquitoes. Adult mosquitoes can feed at the canopy level of trees or at ground level. Females are hematophagous and can feed on different hosts, such as birds, reptiles, and mammals (Alencar et al., 2016; Ribeiro et al., 2020). These feeding heights, as well as mosquito species, can influence the composition of the virome (the set of viruses present in a mosquito population).

In general, mosquitoes can act as vectors for different pathogens (protozoa, helminths, and viruses), causing diseases in humans and animals. This can have an impact on public health (Brady and Hay, 2020; Marceló-Díaz et al., 2022). The family Culicidae contains approximately 3,726 species, classified into two subfamilies, Culicinae and Anophelinae (Harbach, 2013). This diversity showcases the complexity of these organisms. Mosquitoes of the genus *Culex* (*Cux*.) are responsible for transmitting the *West Nile virus*, *encephalitis B virus*, *Japanese encephalitis virus*, and *Sindbis virus* (Pettersson et al., 2019). Meanwhile, species such as *Culex (Cux.) chidesteri, Culex (Cux.) renatoi*, and *Culex (Cux.) quinquefasciatus* have been described and found to be infected with the *Oropouche virus*, and can also be vectors for bancroftian filariasis (*Wuchereria bancrofti*) and *Saint Louis encephalitis virus* (Forattini, 2002; Harbach, 2013). Mosquitoes of the subfamily Anophelinae also transmit malaria parasites. The subgenus *Nyssorhynchus* (*Nys*.) includes the species *Anopheles (Nys.) strodei*, which is recognized as an important vector of the disease, for example malaria (Root, 1926; de Oliveira-Ferreira et al., 1990; Conn et al., 2002; Duarte et al., 2013). These mosquito species, coupled with anthropogenic factors such as urbanization and global warming, contribute to increasing mosquito populations and the risk of disease transmission, making vector control and pathogen monitoring essential, especially for viruses that are public health priorities (Rocklöv and Dubrow, 2020).

In this scenario, the importance of metagenomic approaches to mosquitoes stands out as a tool to assist virological surveillance in identifying known or unknown viruses associated with diseases transmitted by hematophagous mosquitoes (Hameed et al., 2020; Oguzie et al., 2022; Mirza et al., 2024). Between 2016 and 2022, there has been an increase in mosquito virome studies, resulting in the discovery and description of over 2,000 new virus species (Li et al., 2015; Ghurye et al., 2016; Shi et al., 2016; Sadeghi et al., 2018; Wu et al., 2020; Feng et al., 2022).

Zoos are a potential hotspot for zoonotic disease because they harbor species of animals from different continents or biomes. Daily care activities, veterinary procedures, and interactions between animals and zoo personnel or visitors inevitably lead to direct and/or indirect contact between species that would not typically occur in their natural environments. Recent reports from several zoos have highlighted cases of SARS-CoV-2 infections in carnivores, artiodactyls, and non-human primates, which are likely the result of transmission from humans (Cui et al., 2022). Additionally, synanthropic vertebrate species, such as the red fox (*Vulpes vulpes*), rodents, and urban avian populations, are naturally attracted to zoos due to the availability of food and shelter. This situation has created potential transmission routes for various viruses, including poxviruses, avian influenza, and herpes viruses, from urban wildlife to rare exotic animal species housed in zoos (Kurth et al., 2008; Greenwood et al., 2012; Nolting et al., 2013). However, even with this increase in studies on the virome of arbovirus vector mosquitoes, data are still limited in zoological settings. A recent study revealed the intimate relationships among mosquitoes, hosts, and viruses within the São Paulo Zoo, indicating potential interactions in sylvatic environments before pathogens emerge in urban areas (Mirza et al., 2024). Therefore, this study aided in investigating the depth-in mosquito virome at the Sao Paulo Zoo, from mosquito wild to assess the stability of virome composition at different trap heights, collection sites, and mosquito species host, aiming for a better understanding of viral diversity and pathogen-host (mosquito-virus) interactions.

## 2 Materials and methods

### 2.1 Study sites and sample collection

Mosquitoes were collected at the Coordenadoria de Fauna Silvestre (ex-São Paulo Zoological Park Foundation), situated in São Paulo/SP - Brazil. The São Paulo Zoo, located in the southern region of the city, is the largest zoological park in Brazil, encompassing an area of approximately 824,000 square meters of preserved Atlantic Forest. This extensive green space includes the headwaters of the Ipiranga Stream and houses thousands of animals across various species, distributed in enclosures designed to replicate their natural habitats. The zoo is surrounded by residential neighborhoods that display mixed urban characteristics. Some of these neighborhoods are peaceful and well-developed, with adequate infrastructure, while others face urban challenges, such as insufficient maintenance and limited services.

**Figure 1:**
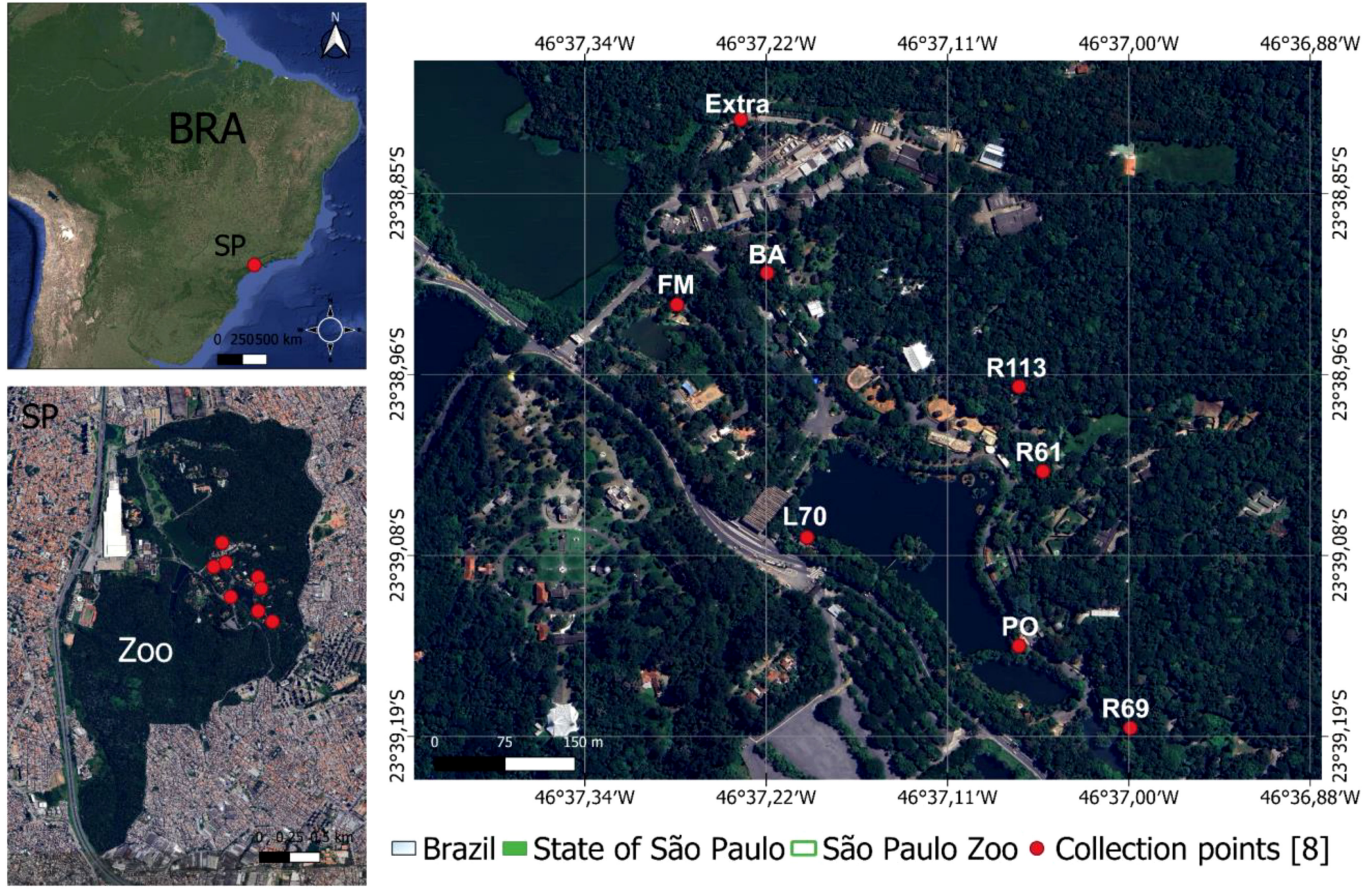
Collection sites and location of Sao Paulo Zoo, Brazil. The R69, R61, FM and BA sites are near different bird enclosures; the PO, L70 and Extra are close to park buildings; R113 is an area near to the raptor enclosure.

The collections took place during several periods: May 5th to 7th, 2020, October 6th to 9th, 2020, November 3rd, 2020, and December 1st, 2020. Sixteen traps utilizing light attraction and a carbon dioxide attractant were positioned at 8 points dispersed throughout the park, mirroring the locations used by Guimarães et al. (2021) (**Figure 1**). The collection sites are separated by a maximum distance of 1000 meters and exhibit the following characteristics: the site R69 (“Recinto 69”) (46°37′0.95″ W, 23°39′11.89″ S) is near a small lake and an enclosure housing various bird species; site L70 (“Lake 70”) (46°37′7.46″ W, 23°39′6.95″ S) is positioned along the shoreline of the larger lake, near the park boundary; site Extra (46°37′14.00″ W, 23°38′49.00″ S) is the sole sampling site outside the park’s visitor area, located close to several buildings; site BA (46°37′13.97″ W, 23°38′53.00″ S) lies within the “Bosque das Aves” (Bird Forest) zoo area, hosting numerous bird species; site FM (“Flamingo Recinto”) (46°37′15.00″ W, 23°38′56.00″ S) is in the zoo’s visitation zone, adjacent to a flamingo enclosure; site R113 (“Recinto 113”) (46°37′3.00″ W, 23°38′57.00″ S) is a diverse raptor display area within the visitor section; and site R61 (“Recinto 61”) (46°37′3.18″ W, 23°39′1.41″ S) features a variety of bird families in its visitation area.

Each point was equipped with 2 traps set at different height [ground (1.5 m off the ground) and canopy height (6 to 10 m off the ground)], employing varying light (white or ultraviolet) and carbon dioxide attractants. The traps remained active for 12 hours between dusk and dawn. Captured insects were carefully placed in labeled cryotubes, promptly frozen alive using liquid nitrogen, and then transported to the laboratory for storage in a -80°C freezer. Detailed information regarding each collection, such as the employed technique, climatic conditions, location, date, quantity of tubes, and collection duration, was meticulously documented in specific reports.

The adult non-engorged females were morphologically identified on a cold table at -20°C using a stereoscope, employing taxonomic keys proposed by Forattini (2002); Consoli and Lourenço de Oliveira (1994), and Lane (1953) at the Entomology Laboratory of Pasteur Institute of São Paulo. Pools of up to 10 mosquitoes were prepared according to species, date, point, and collection site. Specimens with morphological damage, where only identification by gender was possible, were processed individually (**Table 1**).

**Table 1:** Overview of the mosquito pool utilized for virome characterization at the Coordenadoria de Fauna Silvestre (ex-São Paulo Zoological Park Foundation).

Pool	Mosquito species	N	Trap height	Collection site
Meta 20	*Anopheles (Nys.) strodei*	23	Ground	Mix
Meta 21	*Anopheles (Nys.) strodei*	22	Canopy	Mix
Meta 22	*Culex (Cux.) chidesteri*	50	Ground	Extra
Meta 23	*Culex (Cux.) chidesteri*	50	Canopy	Extra
Meta 24	*Culex (Cux.) chidesteri*	20	Ground	R113
Meta 25	*Culex (Cux.) chidesteri*	21	Canopy	R113
Meta 26	*Culex (Cux.) chidesteri*	50	Ground	Flamingos Enclosure (FM)
Meta 27	*Culex (Cux.) chidesteri*	50	Canopy	Flamingos Enclosure (FM)
Meta 28	*Culex (Cux.) renatoi*	21	Canopy	Flamingos Enclosure (FM)
Meta 32	*Aedes (Och.) scapularis*	22	Mix	Mix
Meta 33	*Culex (Cux.) quinquefasciatus*	20	Mix	Mix
Meta 34	*Culex (Cux.)* sp.	26	Ground	Flamingos Enclosure (FM)
Meta 35	*Culex (Cux.)* sp.	47	Canopy	Flamingos Enclosure (FM)
Meta 36	*Culex (Cux.)* sp.	38	Ground	R61
Meta 37	*Culex (Cux.)* sp.	38	Canopy	R61
Meta 38	*Culex (Mel.)* sp.	50	Ground	Extra
Meta 39	*Culex (Mel.)* sp.	50	Canopy	Extra
Meta 40	*Culex (Cux.) chidesteri*	20	Ground	Bosque das Aves (BA)
Meta 41	*Culex (Cux.) chidesteri*	28	Canopy	Bosque das Aves (BA)
Meta 42	*Culex (Cux.) chidesteri*	22	Ground	R61
Meta 43	*Culex (Cux.) chidesteri*	35	Canopy	R61
Meta 44	*Culex (Cux.) chidesteri*	28	Ground	Lake 70 (L70)
Meta 45	*Culex (Cux.) chidesteri*	26	Canopy	Lake 70 (L70)
Meta 46	*Culex (Cux.) chidesteri*	26	Ground	R69
Meta 47	*Culex (Cux.) chidesteri*	33	Ground	R69
Meta 48	*Culex (Cux.) chidesteri*	50	Canopy	Flamingos Enclosure (FM)

This study was carried out according to the Ethical Principles in Animal Research and was approved by the Ethics Committee of the Institute of Tropical Medicine, University of São Paulo (CPE-IMT/398A, 8 February 2019), as well as the Brazilian Ministry of Environment (SISBIO 67527–4, 12 April 2022).

### 2.2 RNA extraction and sequencing

The insects were grouped into pools containing between 20 and 50 specimens according to morphological identification, location and collection date. Each pool was homogenized in a FastPrep-96 homogenizer (MP Biomedicals, USA) for 1 min at 1500 rpm using 900μL of Hanks’ buffered salt solution (HBSS) and 1g of ceramic beads, followed by centrifugation at 14,000 rpm for 10 minutes. Approximately 300 µL of the supernatant was filtered through a 0.45 μm filter (Merck Millipore, Billerica, MA, USA). The filtrate was subjected to treatment with a mixture of nuclease enzymes (7 µL of TURBO DNase and 3 µL RNase Cocktail Enzyme Mix, Thermo Fisher Scientific, Waltham, MA, USA) in order to digest unprotected nucleic acids. RNA extraction was performed with Maxwell 16 Viral Total Nucleic Acid Purification kit (Promega, Inc., Madison, WI, USA) according to the manufacturer’s instructions and cDNA synthesis was performed with SuperScript IV (Thermo Fisher Scientific, Waltham, MA, USA). A second strand of cDNA synthesis was performed using DNA Polymerase I Large (Klenow) Fragment (Promega Inc., Madison, WI, USA). The DNA library was prepared with the Nextera XT DNA library preparation kit (Illumina Inc., San Diego, CA, USA) according to the manufacturer’s instructions. Sequencing was carried out on MiSeq Sequencer (Illumina Inc., San Diego, CA, USA) at the Central Laboratory of the Hospital das Clínicas of the University of São Paulo.

### 2.3 Virome characterization

The sequenced reads were firstly quality checked using FastQC tool followed by trimming of low quality reads using Trimmomatic (Bolger et al., 2014). Next, virome assemblies were generated using three different assembly tools: rnaviralSpades (Bushmanova et al., 2019), metaSpades (Nurk et al., 2017) in default mode and MEGAHIT in default mode. All contigs sequences obtained from the three different assembly tools described above were clustered using the CD-HIT tool in order to exclude redundant sequences having >= 98% of identity. Additionally, the contigs with a length < 600bp were removed. The sequenced contigs were taxonomic classified using DIAMOND (Buchfink et al., 2015) performing a blastx search against the retrieved viral proteins; E-value cutoff was set at 1E-5 to maintain high sensitivity and low false-positive rate. Only contigs where the first 5 hits were of viral origin were kept for further analysis. Following BLAST analysis, accession numbers were parsed to NCBI taxonomy files in R using package “taxonomizr” and if the contigs showed the multiple species, they were annotated to the last common ancestor (LCA). The compute abundance of each contigs, the trimmed reads were mapped against all contigs using Bowtie2 in “end-to-end” sensitive mode to prevent false-positive mapped. The host predicted for virus species were obtained from viral genome sequences available from GenBank via NCBI Virus repositories (Bethesda, 2004).

### 2.4 Ecological data analysis

The overall virome composition profile was examined using alpha-diversity metrics, focusing on richness, abundance and Shannon (H). The virus species level obtained from the taxonomy binning served as input for the alpha-diversity metrics analysis, conducted using Vegan R (Oksanen et al., 2008). The overall significance levels for each factor—i.e. trap height, host species, and collection—were evaluated using analysis of variance (ANOVA) and dunn test *post-hoc*.

Next, to evaluate the virome community structure between samples, principal component analysis (PCA) and non-multidimensional scaling (NMDS) were generated. The Vegan package was used to compute NMDS and used to produce the ordination plots, in which each source was represented by specific icons with the Bray–Curtis set as the matrix distance. Abundance count data were normalized following the normalization method provided in the phyloseq (McMurdie and Holmes, 2013) and metagenoSeq (Paulson et al., 2013) packages.

All statistical analyses were conducted using R version 4.0.1 within RStudio. Graphs were generated using the pheatmap (Kolde, 2019), networkd3, treemap (Gandrud, 2020), ggVennDiagram (Gao et al., 2024) and the ggplot2 (Ito and Murphy, 2013) packages in R.

## 3 Results

### 3.1 Overview virome

**Figure 2:**
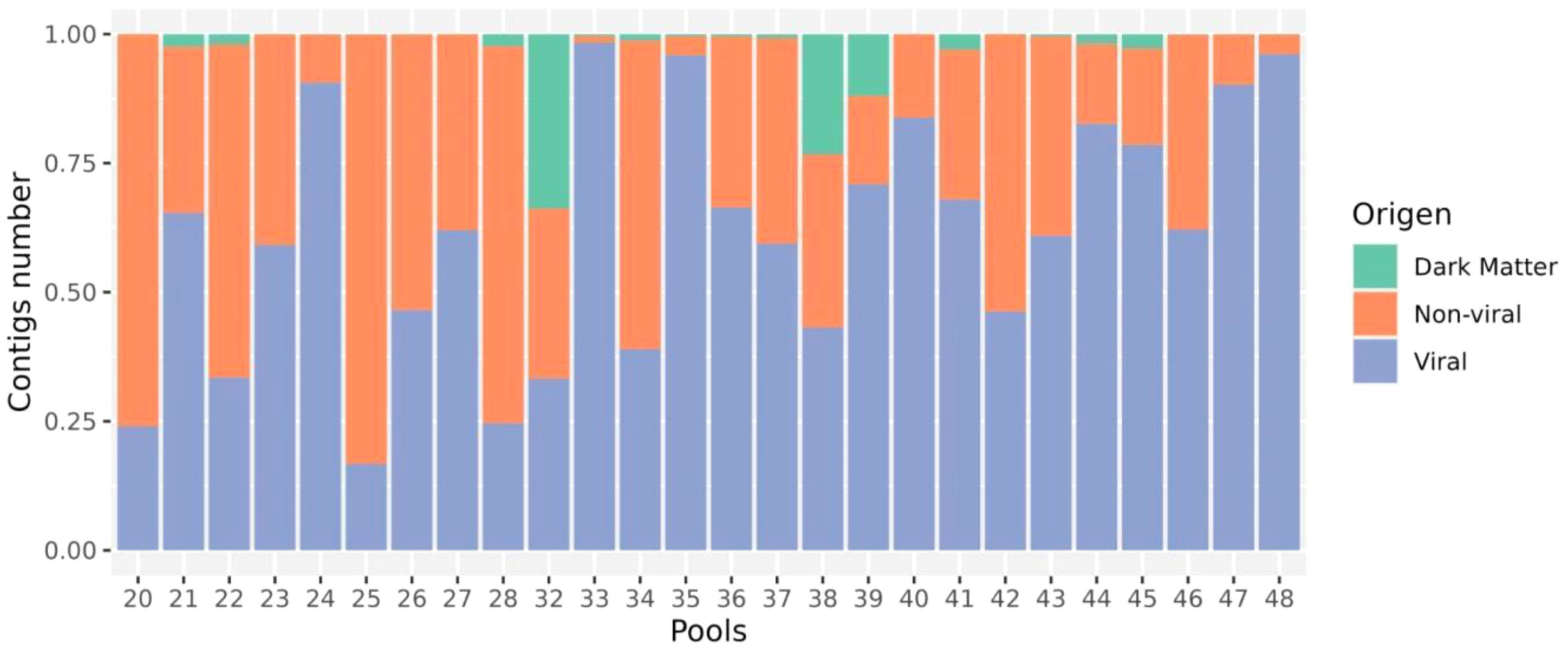
Relative composition of viral, host (non-viral) and dark matter (no match) reads in every library.

Virome sequencing of twenty-six pools (from a total 886 mosquitoes) resulted in an average of 929,615 (range 179,549 - 1,648,206) reads per pool, assembled *de novo* into 61–7,074 contigs for virus discovery and characterization (**Supplementary Table S1**). From a total of 28,204 contigs assembled, we identified 1,039 contigs as viral, 24,822 as non-viral (including eukaryotic and prokaryotic matches), and 2,343 were unclassifiable and did not match any sequences according to DIAMOND Blastx against the NR NCBI database (available for download on 2023-07-28). The proportion of reads mapped to viral sequences compared to the total number of non-viral reads per pool varied between 16.7%–98.3% (**Figure 2**). The pool 20, 22, 28 and 32 were characterized by a lower number of viral reads (<30%) to non-viral reads. Notably, three pools (32, 38 and 39) were characterized by a higher proportion of unclassifiable reads (33.7%, 23.9% and 11.9%, respectively).

**Figure 3:**
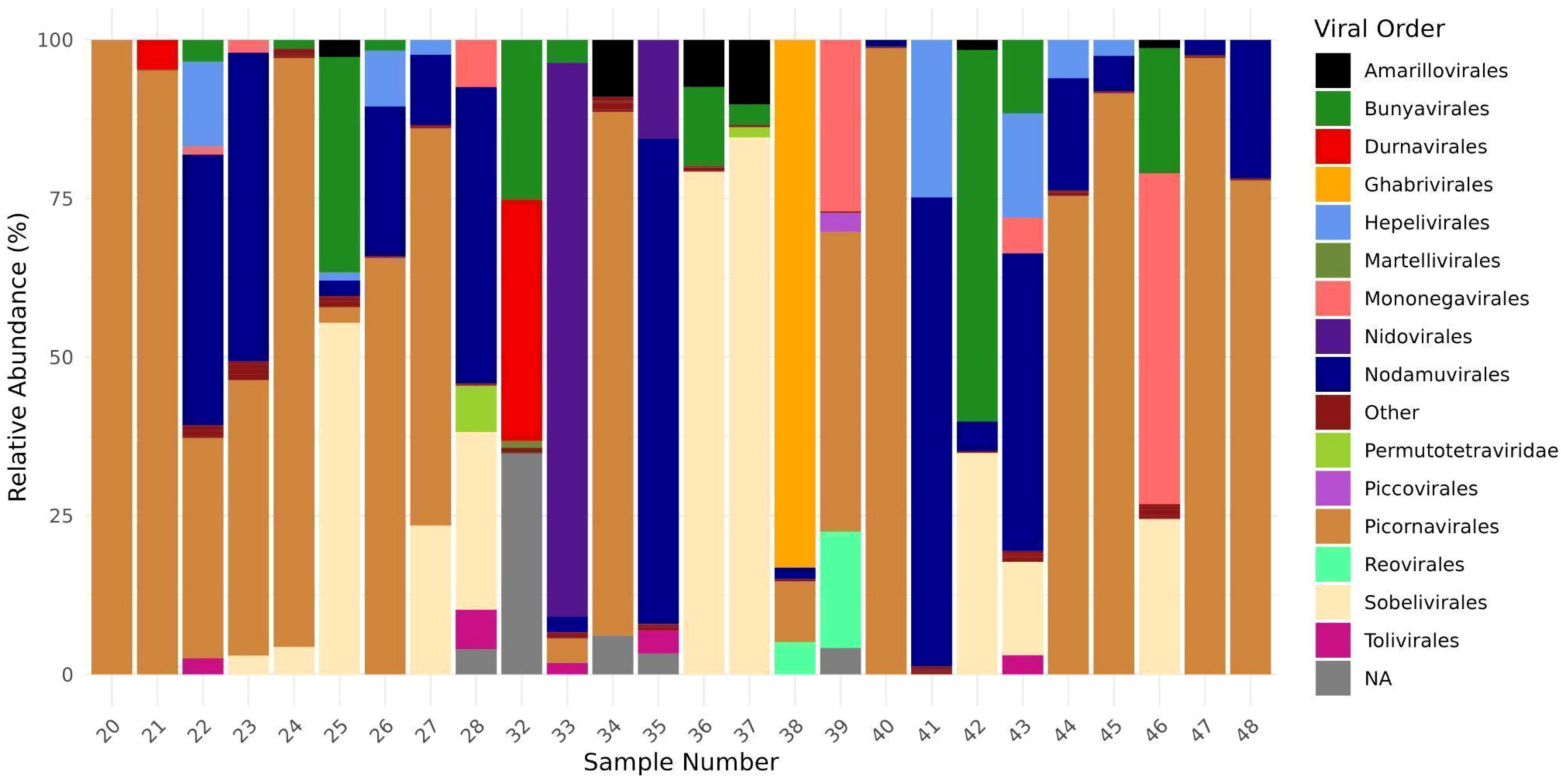
Relative abundance of viral order from twenty-six mosquitoes pools. The abundance of viral families was estimated computing the number each viral group divides by total viral reads from each pool.

**Figure 4:**
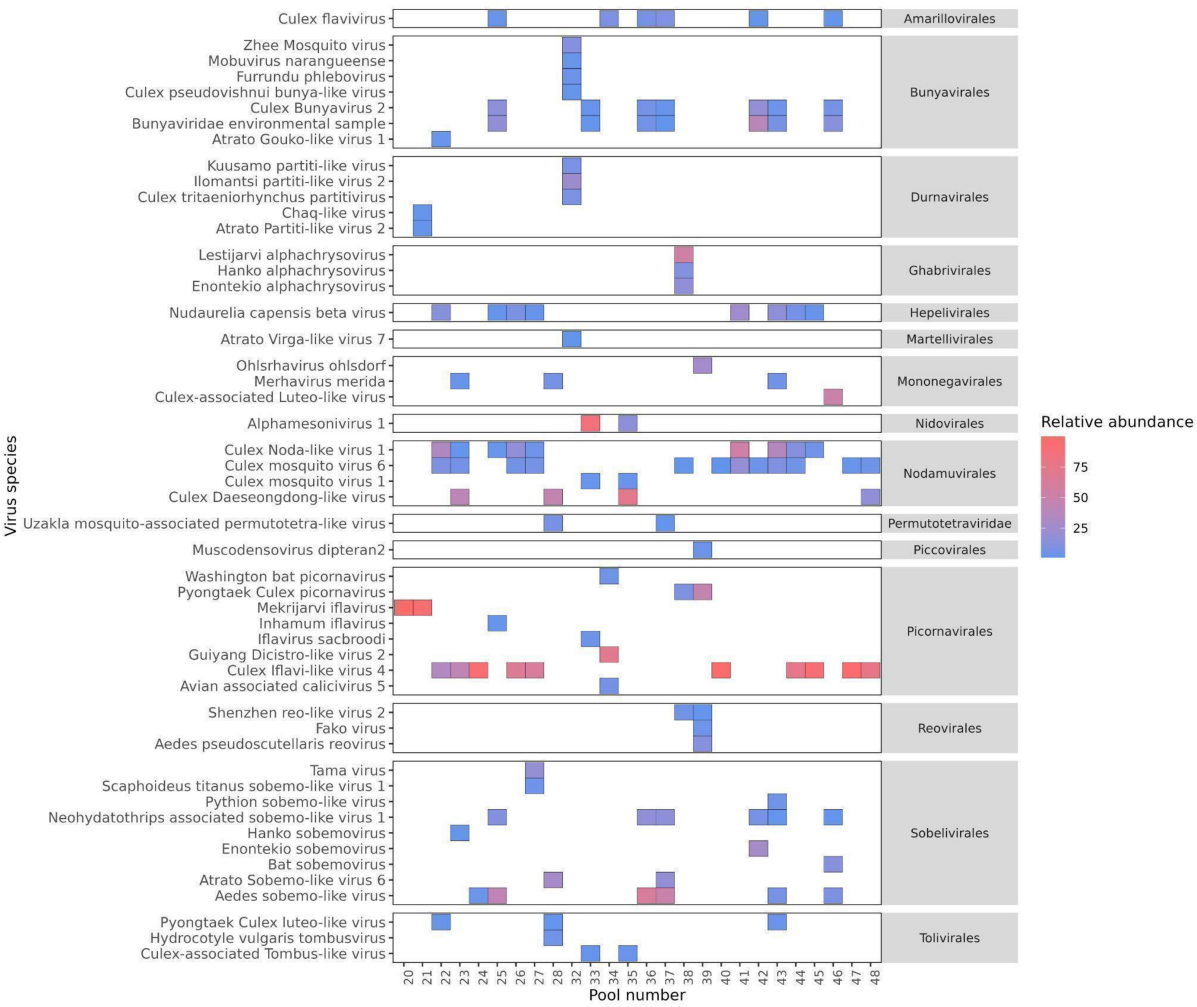
The prevalence and abundance of viral species with > 1% relative abundance among libraries. The viruses are grouped according to the order in which they were predicted to be classified. The absence of viruses is indicated by blank spaces, while the color bar illustrates the relative abundance of each virus in comparison to all reads within each library.

The identification of 1,039 contigs as viral revealed a large diversity of viruses, comprising twenty-two order, third-six families, forty-six genera, and two hundred and thirty-nine species (**Supplementary Table S3**). Furthermore, taxonomic information at the class, order, family, or genus level could not be retrieved for 70 viral sequences, which were assigned as Metagenomic Assembled Sequences (MAGs) in the NCBI database. These include *Mika virus* (MW434952), *Hubei mosquito virus 3* (OL700061), *Hubei virga-like virus 2* (MW435006), *Canya virus* (MW434766) and other 19 taxa (**Supplementary Table S2**). Based on individual abundance, the predominant sequences were associated with taxa known to infect insects, notably the orders *Picornavirales*, *Nodamuvirales*, and *Sobelivirales*, which exhibited high abundance. Each of these orders was primarily represented by a single family (**Figure 3**), in which *Picornavirales* and *Nodamuvirales* were each detected in 17 pools, while *Sobelivirales* were detected in 10 pools. Only relative abundance value > 1% per pool was retained (**Figure 4**). In the order *Picornavirales*, *Culex Iflavi-like virus 4* was found to be widely distributed and abundant within the pools, being present in 10 out of 26 samples, with a prevalence of over 50%, and *Mekrijarvi iflavirus* was highly abundant (>70 percent of total reads) in two pools. The order *Nodamuvirales* was represented by four viruses, among which the most prevalent were *Culex mosquito virus 6*, identified in 12 samples, and *Culex Noda-like virus* 1, identified in 9 samples. The order *Sobelivirales* exhibited the highest diversity, with a total of twelve viruses identified.

### 3.2 Alpha diversity

**Figure 5:**
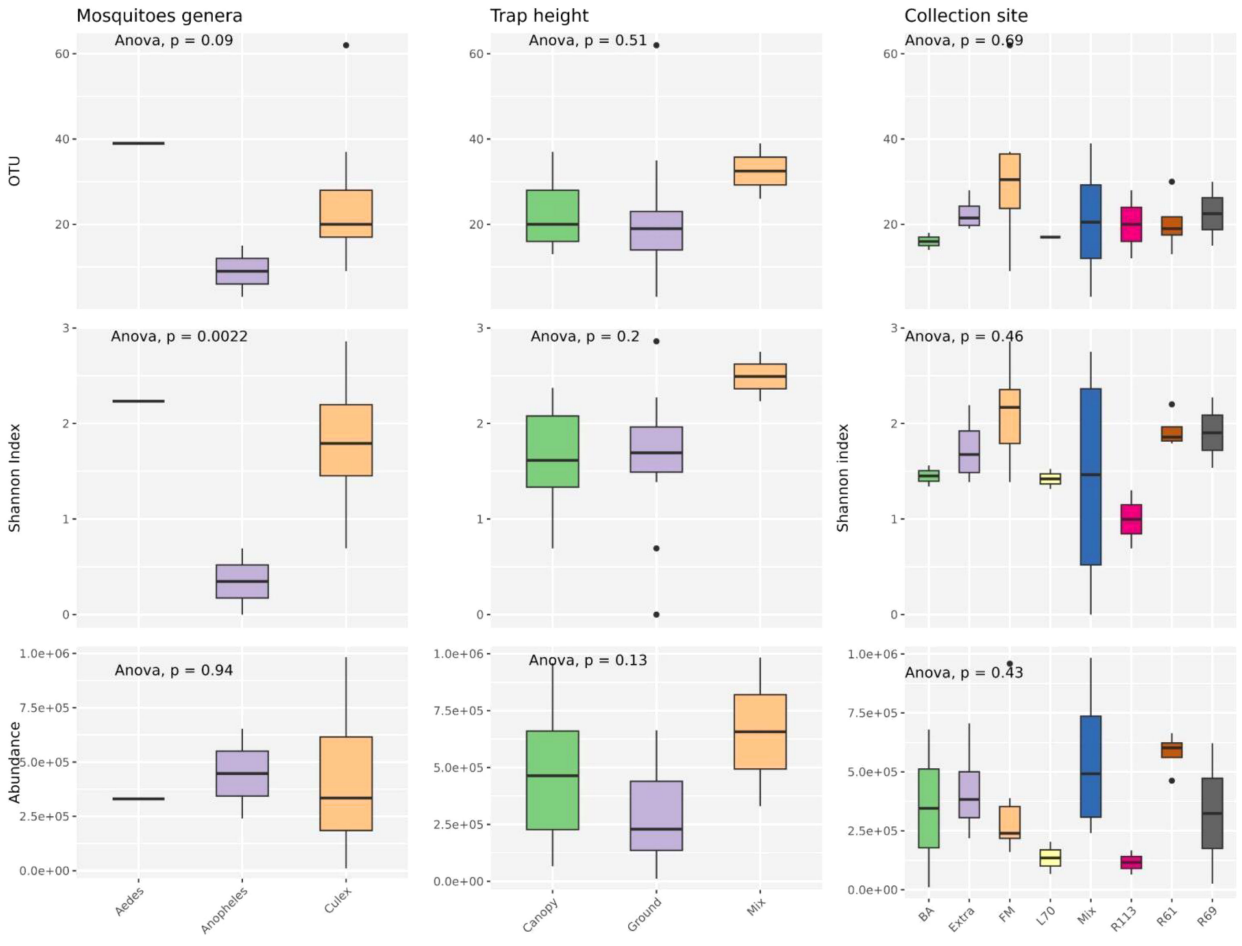
Analyses were conducted to assess the variations in observed richness (OTU), alpha diversity (Shannon index) and abundance (RPM) across various mosquito species, trap level and collection sites categories. An ANOVA test examined differences among variables in each category. P-values are displayed at the top of each graph. Boxplots depict the median with bold lines, while the upper and lower hinges represent the first and third quartiles, respectively. Different ecological groups are represented by distinct colors.

The alpha diversity of mosquito-associated microbial communities, measured by operational taxonomic unit (OTU) richness, Shannon diversity index, and microbial abundance, showed distinct patterns across mosquito genera, while trap height and collection site had less pronounced effects (**Figure 5**). OTU richness, which represents the number of unique microbial taxa, was highest in *Culex* mosquitoes, followed by *Anopheles* and *Aedes*, although these differences were not statistically significant (*p* = 0.09). Shannon index, which accounts for both richness and evenness, displayed significant variation among mosquito genera (*p* = 0.0022), with *Culex* showing a more diverse and balanced microbial community. This suggests that *Culex* mosquitoes might either encounter or tolerate a broader range of microbial taxa due to their ecological or physiological traits. Microbial abundance, reflecting the total microbial load, was consistent across genera, with no significant differences detected (*p* = 0.94). When examining trap height (canopy, ground, and mixed), no significant differences were observed in OTU richness (*p* = 0.51), Shannon index (*p* = 0.20), or microbial abundance (*p* = 0.13). This indicates that the vertical stratification of sampling had minimal influence on the diversity and composition of mosquito-associated microbiota, suggesting that microbial acquisition and maintenance are not strongly dependent on vertical spatial factors. Similarly, collection site did not significantly affect alpha diversity. OTU richness (*p* = 0.69), Shannon index (*p* = 0.46), and microbial abundance (*p* = 0.43) were comparable across the different sampling locations. This consistency across sites implies that microbial communities associated with mosquitoes are relatively resilient to geographical and environmental variation, at least within the scope of the sampled locations.

Overall, these findings highlight that mosquito genus exerts a more substantial influence on microbial diversity, particularly on community evenness, compared to environmental variables such as trap height or collection site. This may reflect intrinsic biological differences among mosquito genera that shape their interactions with microbial communities.

### 3.3 Beta diversity

**Figure 6:**
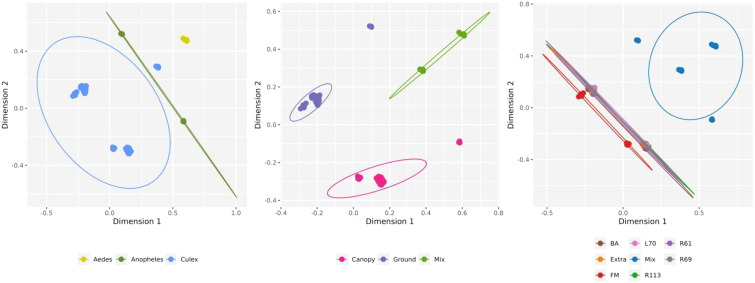
Non-metric multidimensional scaling (NMDS) for viral composition on viral species level by host genera, trap height and collection sites. Circles show 95% normal probability ellipse for each species group (bottom panel).

After assessing the global pattern of mosquito-associated microbial communities, we further analyzed differences in their composition using beta diversity. Bray-Curtis dissimilarities, calculated from microbial abundance data, were used for non-metric multidimensional scaling (NMDS) to visualize the relationships between samples (**Figure 6**). Our results revealed that microbial communities were primarily structured by mosquito genera rather than by trap height or collection site. Each genus (*Aedes*, *Anopheles*, and *Culex*) formed distinct clusters in the ordination space, indicating significant differences in microbial composition. The NMDS analysis of trap height shows some separation of canopy traps, while ground and mixed traps exhibit slight overlap, suggesting moderate vertical structuring of microbial communities. Collection sites, however, showed greater overlap, with no distinct clustering patterns except for the site labeled “Mix,” which formed a relatively isolated group. Moreover, the different mosquito species from the same genera grouped in the same cluster, suggesting a close virome composition between them (**Supplementary Figure S1**). These findings suggest that while mosquito genus exerts a strong influence on microbial composition, environmental factors such as trap height and collection site contribute less to the observed beta diversity.

**Figure 7:**
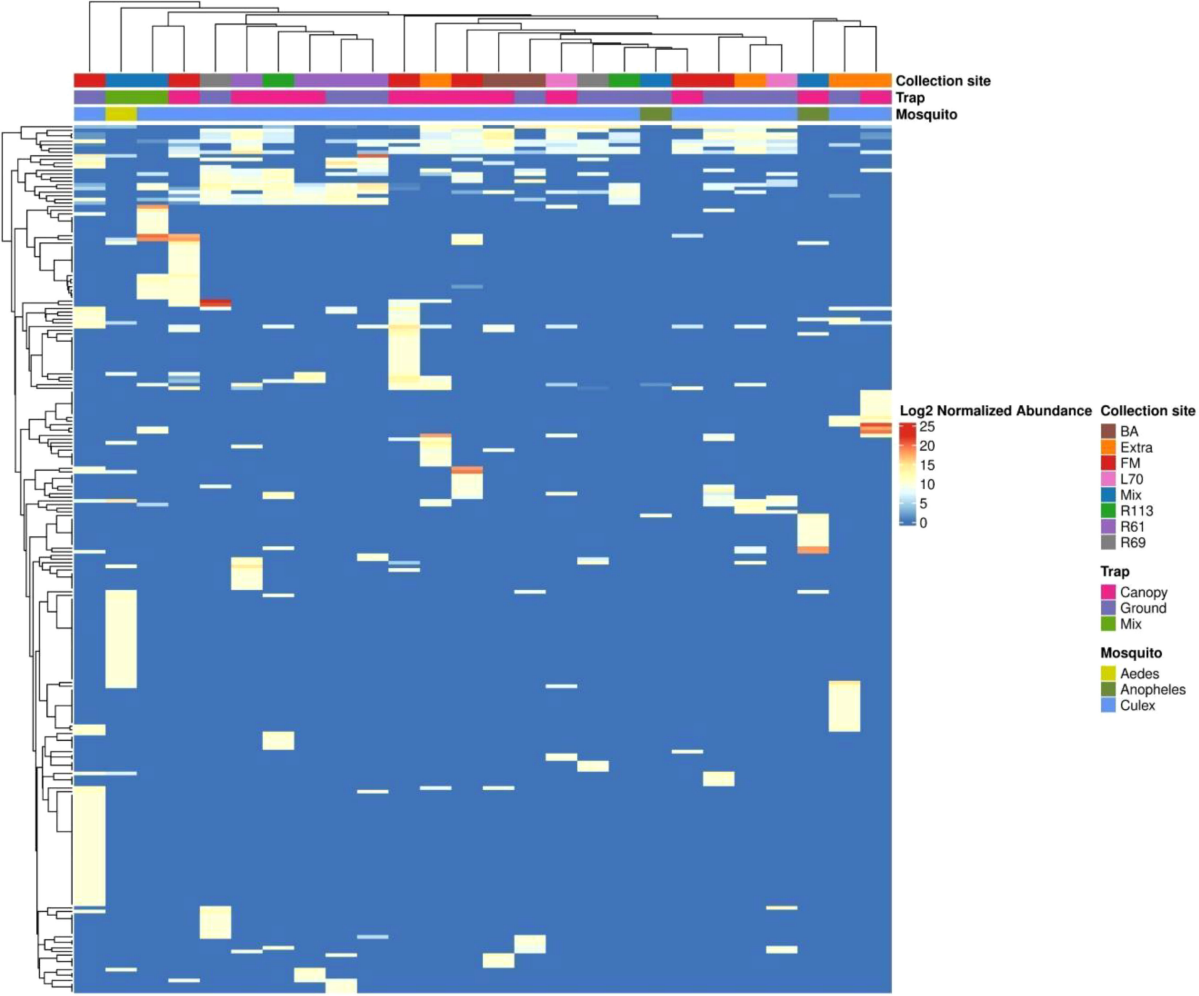
The heatmap displays the normalized abundance of 229 viral species detected across the libraries. Normalized read counts, processed through metagenomeSeq and presented on a log2 scale, are depicted. Hierarchical clustering is conducted using the Euclidean distance matrix derived from these normalized read counts.

The lack of virome structure associated with collection site and trap height was particularly evident in the heatmap, complemented by an abundance-based dendrogram analysis (**Figure 7**). For this analysis, only viruses contributing at least 1% of the total relative abundance were included, narrowing the selection to 56 viral species out of the 229 previously identified across the 26 mosquito pools. This filtering criterion ensured that the most ecologically and biologically significant viral species were represented. Despite this focused approach, the clustering patterns failed to show any clear associations with collection site or trap height. However, distinct clustering was observed within viromes based on mosquito genera, with *Aedes*, *Anopheles*, and *Culex* each forming separate clusters. This suggests that mosquito host identity, rather than environmental factors, is the primary driver of virome composition in this dataset.

### 3.4 Co-occurrence

**Figure 8:**
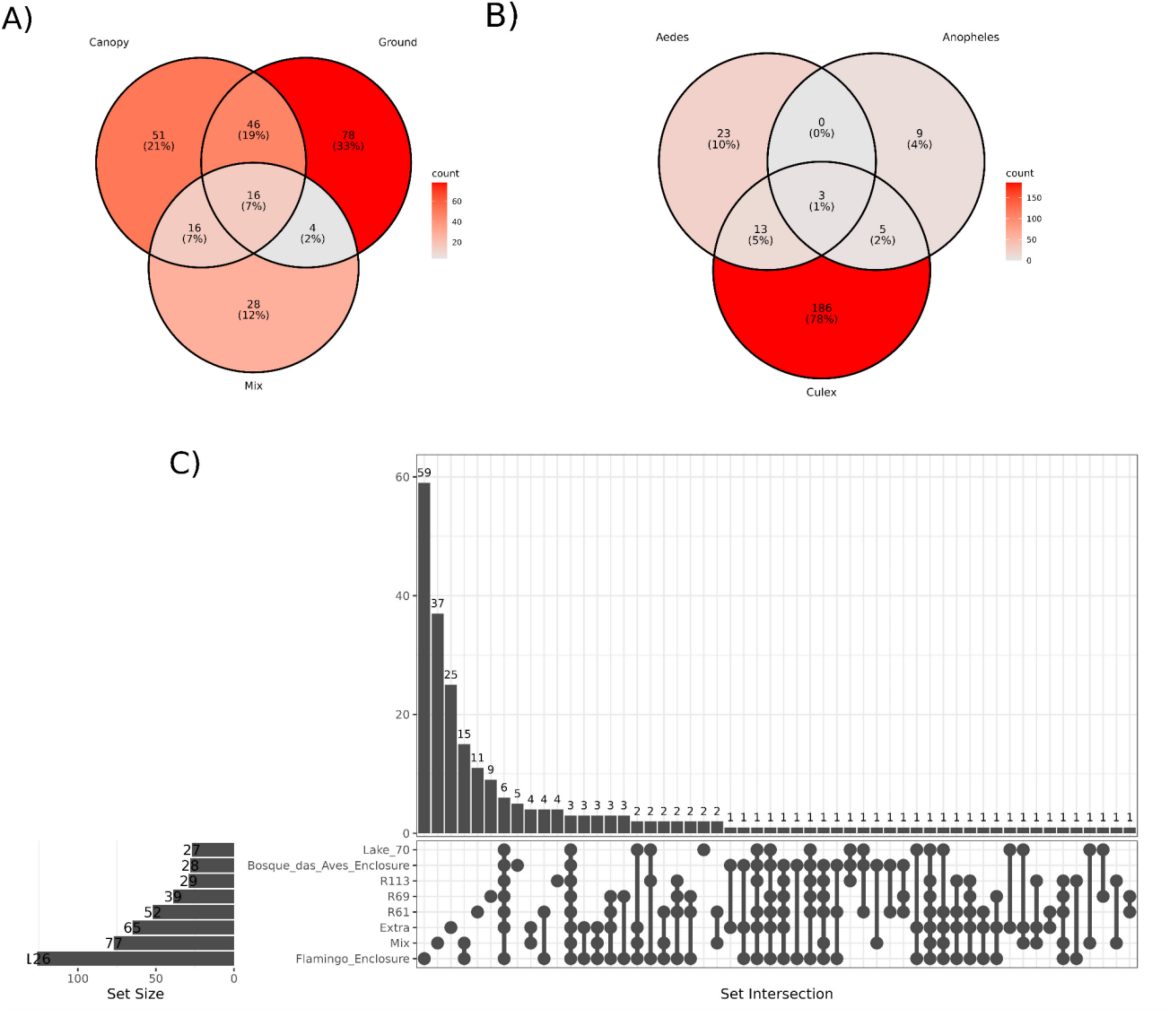
Representation of virus species shared between different trap height **(A)**, host genera **(B)** and sampling locations **(C)**. The intensity of the color represents is proportional to the percentage of viral species present in each group.

To evaluate the shared taxa among mosquito hosts, collection sites, and trap height, we conducted a co-occurrence analysis of viral species (**Figure 8**). Across different trap heights, we noted that the ground trap had the highest number of identified viral species (144), followed by the canopy trap (129) and the mixed trap (64). Out of the 157 viral species identified (66%), each were exclusively present in a single trap. Conversely, 82 species (34%) were detected in at least two different traps, with the most common co-occurrence observed between the canopy and ground traps, comprising 46 identifications (19%) (**Figure 8A**; **Supplementary Table S4**).

Concerning mosquito hosts, 218 (92%) viral species were exclusively associated with a single host, while only 21 (8%) were detected across multiple hosts. Notably, the most prevalent co-occurrence was observed between *Culex* and *Aedes*, with 13 viral species exclusively co-occurring between them. Conversely, no shared viral species were identified between *Anopheles* and *Aedes* in this study (**Figure 8B**; **Supplementary Table S5**).

When examining the viral species identified at each collection site, it became evident that 150 (62%) viral species were exclusively present at one site, while 89 species (38%) were found across multiple locations. The Bosque das Aves enclosure (BA) site shared the least viral contigs with any other site (23 in total), whilst the Flamingo enclosure (FM) site, although different ecological features, shared more viral contigs in common with R61 sites (67 contigs) (**Figure 8C**; **Supplementary Figure S2**, **Supplementary Table S6**). A notable number of intersections among the various locations highlights a substantial overlap of the mosquito virome across the different areas of the São Paulo Zoological Park, and this may reflect similarities between these habitats.

### 3.5 Host-virus dynamics

**Figure 9:**
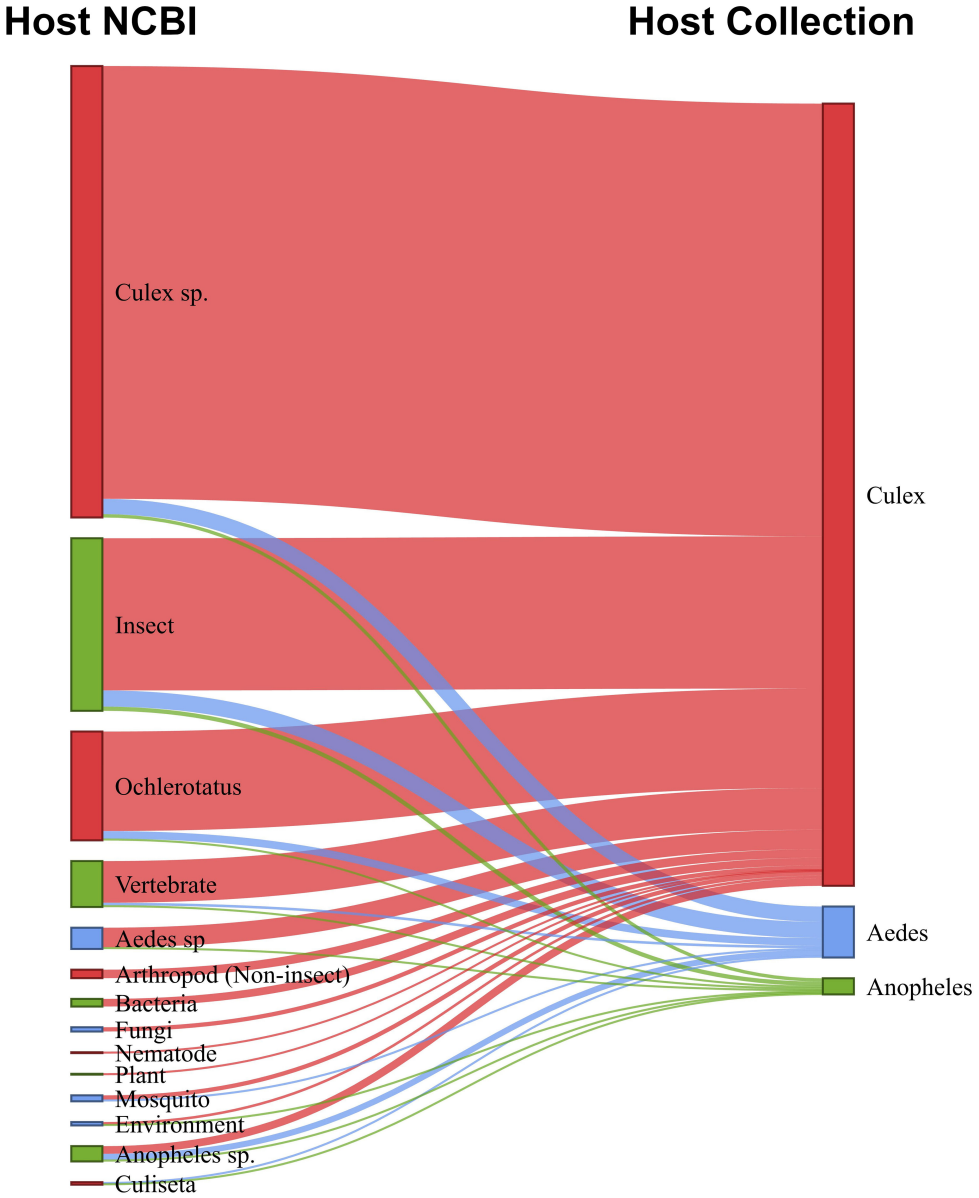
Sankey diagram shows relationship between host-virus records in the NCBI database (left) and mosquito source of virus in this study (right).

Based on a combination of our data and virus records from the NCBI database, we constructed a network to visualize the associations between known viruses host and mosquito species collection, and their potential non-mosquito hosts, including animals and plants (**Figure 9**; **Supplementary Table S7**). For convenience, we highlighted the main host groups. For example, within the arthropod group, insects were shown separately, and among the insects, mosquitoes were further differentiated. Additionally, some mosquito genera, such as *Culex*, *Anopheles*, *Aedes*, *Culiseta*, and *Ochlerotatus*, were specifically identified. Our findings indicated that mosquitoes from the *Aedes*, *Anopheles*, and *Culex* genera harbored viruses from a diverse range of host groups, including arthropods (primarily mosquitoes), vertebrates, plants, fungi (mycoviruses), and bacteria (bacteriophages). Insect-specific viruses (ISVs) were the most prevalent across all mosquito species, accounting for 85% of the identified viruses. Several viruses were linked to multiple insect species, suggesting horizontal transmission across different insect hosts. For *Culex* mosquitoes, the majority of known viruses (57%) were exclusively associated with mosquitoes at the genus level. In contrast, for *Anopheles* mosquitoes, only 10% of the known viruses were associated with this host genus and for *Aedes* mosquitoes, none of the viruses found had been previously associated with this host.

A minority of viruses collected from mosquitoes were associated with non-arthropod hosts. Most of these viruses are acquired during the mosquitoes’ feeding, such as plant-associated and vertebrate-associated viruses. Among the vertebrate viruses, we highlighted the presence of viruses known to infect bats, birds, cattle, horses, and monkeys, suggesting possible food sources for the mosquitoes. Another portion of these viruses originates from parasites in the mosquito’s non-viral microbiota, including bacteriophages, fungi, and other organisms. These findings are supported by the analysis of the abundance of non-viral reads (**Supplementary Figure S3**).

## 4 Discussion

In this study, we used a viral metagenomic approach to evaluate the virome composition from wild mosquitoes collected in the São Paulo Zoo. We identified 229 virus species through total RNA-sequencing of 866 mosquitoes collected from three genera: *Culex*, *Anopheles* and *Aedes*. These mosquitoes were chosen because they are vectors of important virus, such as Dengue, Chikungunya and Zika virus. Furthermore, we selected only adult non-engorged female mosquitoes to maximize the ecological relevance of insect-virus interactions, striking a balance between cost-effectiveness and sequencing depth. The ecological aspects of the mosquito-virus-environment relationship were explored, revealing a virus structure that is widely distributed throughout different parts of the Zoo and strongly influenced by the host rather than location-specific features.

The study of mosquito viromes has garnered attention due to their potential influence on the transmission dynamics of arboviruses (Hobson-Peters et al., 2013; Kuwata et al., 2015; Nasar et al., 2015; Hall-Mendelin et al., 2016; Schultz et al., 2018; Öhlund et al., 2019). Understanding how ISVs interact with pathogens within mosquito hosts could offer new insights into their role in modulating vectorial capacity. Additionally, spatiotemporal correlations between virome composition and disease outbreaks may elucidate whether ISVs contribute to or mitigate pathogen transmission. Despite this potential, the host range and interspecies transmission capabilities of many ISVs remain largely unexplored. Furthermore, the effects of viral infections on mosquito physiology and development, which could indirectly influence their ability to transmit diseases, are critical areas for future research. For instance, studies on *Aedes* mosquitoes have uncovered ISVs closely aligned with medically significant viruses, which could influence the replication or transmission of arboviruses like dengue and Zika (Souza-Neto et al., 2019). Similarly, viromes of *Anopheles* and *Culex* have revealed ISVs that interact with viral pathogens, potentially altering mosquito physiology or vectorial capacity (Bolling et al., 2012; Urakova et al., 2020). These findings emphasize the intricate relationship between mosquito viromes and pathogen transmission dynamics, underscoring the need for integrated virome studies to unravel their ecological and epidemiological implications.

The virome of mosquitoes from Zoo were dominated by *Picornavirales*/*Iflaviridae*, *Nodomuvirales*/*Nodaviridae*, *Sobelivirales*/*Solemoviridae* order and families, respectively, both in abundance and frequency. The order *Picornavirales* is a small, non-enveloped virus with positive-sense single-stranded RNA genomes and includes various families that infect plants, marine organisms, and animals (Valles et al., 2017). Some families can infect mosquitoes, such as *Dicistroviridae* and *Iflaviridae* (*Iflavirus*), which have been identified in *Culicoides* (Shi et al., 2015; Yang et al., 2023). The order *Sobelivirales* is single-stranded, positive-sense RNA genomes (ssRNA+) virus, infecting mainly plants and causing a variety of plant diseases, as well as viruses that infect mosquitoes. The order comprises three families: *Solemoviridae*, *Barnaviridae*, and *Alvernaviridae*, distributed across two subfamilies, seven genera, and 122 species (Nga et al., 2011; Sõmera et al., 2021; Thomas et al., 2021; Adegbola et al., 2022; Guo et al., 2022). Finally, the *Nodaviridae* family primarily infects insects in nature, as well as larvae and juvenile fish. It consists of two genera: *Alphanodavirus* and *Betanodavirus* (Sahul Hameed et al., 2019; Johnson et al., 2001). Therefore, the mosquito virome is composed of viral groups closely related to insects or organisms they feed on, such as plants (in their adult phase), and also aquatic animals with which insects interact during the larval stage, highlighting the interconnection between different environments and ecosystems.

Our research revealed a rich diversity within the virome of the three mosquito vector genera, encompassing viruses associated with plants, fungi, bacteria, vertebrates, algae, and protists. Although all samples were collected from adult mosquitoes, we also detected green algae (from the orders *Sphaeropleares* and *Chlamydomonadales)* and mollusca (from the orders Bivalvia and Cephalopod), known to serve as nutritious food sources for mosquito larvae in aquatic environments (Tuno et al., 2006; Cuesta et al., 2021) (**Supplementary Figure S3**). These findings indicate that the virome of mosquitoes collected from natural settings, particularly in tropical regions with minimal human interference, may reflect complex interactions with their environment. This phenomenon is likely influenced by various factors, such as breeding and feeding sites.

Recent studies on the virome have consistently identified stable viral communities within individual mosquito species, a pattern observed across different individuals (Shi et al., 2019, 2020). This stability has also been documented throughout various developmental stages of *Aedes albopictus*, suggesting the possibility of vertical transmission. Based on this stability across species, we hypothesized that similar patterns would be quantitatively reflected in mosquito populations at São Paulo Zoo.

Our findings, however, reveal significant heterogeneity in viral composition among different mosquito genera. This supports the notion that mosquitoes harbor a “core virome”, a group of viruses consistently present across individuals or populations. Most viruses identified in this study exhibited either genus-specificity, being exclusive to a single mosquito genus, or strong host preference. These findings align with the hypothesis of co-evolution between mosquitoes and their associated viruses, suggesting a long-term, adaptive relationship (Cook et al., 2013; Dudas and Obbard, 2015; Marklewitz et al., 2015).

Evidence of such prolonged associations includes studies showing vertical or transovarial transmission (TOT) of insect viruses, with some even integrating into the genomes of their arthropod hosts (Fort et al., 2012; Bolling et al., 2012; Lequime and Lambrechts, 2017). These findings underscore the intricate evolutionary dynamics shaping the relationship between mosquitoes and their viromes.

When we analyzed several species within the *Culex* genus, we observed a significant number of shared viruses among them (**Supplementary Figure S4**, **Supplementary Table S8**). This suggests a reduced intra-genus barrier compared to inter-genus barriers in shaping virome composition among mosquito species. A similar result was observed in previous studies, that mosquito pools within the same genera tend to share a higher number of common viruses (Shi et al., 2020; Pettersson et al., 2019). Given that some of the *Culex* specimens studied belong to the *Culex pipiens* complex (Smith and Fonseca, 2004; Batovska et al., 2016), it is reasonable to expect similarities in their virome composition. Therefore, the similarity observed in viromes among the *Culex* species may be attributed, at least in part, to their close evolutionary relationships. This closeness likely influences similarities in their cellular environment, immunological response, and potentially their ecological niche.

Our findings unveil a uniform virome composition across diverse sites within the Sao Paulo Zoo, despite their environmental disparities. However, our collection sites in São Paulo Zoo are located within a small geographical area, separated by less than 1 km from each other. Considering the limited flight range of mosquitoes, typically limited to about 200 m in their lifetime (Moore and Brown, 2022), the sharing of microbiota among geographically close mosquito populations is favored, whether through horizontal transmission, shared food sources, aquatic environments, vertical transmission, or other means (Agboli et al., 2019). Several studies have highlighted the spatial distribution of mosquito viromes, indicating substantial taxonomic diversity overlap across different geographic scales (Shi et al., 2019; Thongsripong et al., 2021). In a relatively confined area (less than 20 km wide), this composition appears primarily influenced by the taxonomic classification of host mosquitoes within a relatively confined area (all our study sites are within 1 km apart). Viruses, being obligate intracellular parasites, rely on host cells for their replication and function. They must recognize specific host cells, enter them, and manipulate various molecular mechanisms within the host cell to complete their replication cycles. These characteristics inherently restrict viruses to specific host species rather than specific geographic locations. Therefore, it is unsurprising that we observe a pattern of host species specificity rather than location specificity.

The principle described above can also be applied to the virome composition similarity found in mosquitoes collected at traps of different heights. Mosquito flying height is related to host feeding patterns (Lee et al., 2006; Swanson and Adler, 2010), and similar to feeding behaviors, flight altitude can change based on location, season, time of day, and the physiological condition of the mosquito (Snow, 1979; Snow, 1977; Jansen et al., 2009; Swanson and Adler, 2010). While *Culex* species are more likely to be captured in tree canopy traps in contrast to *Aedes* species (Jansen et al., 2009), this is likely related to a preference of certain *Culex* species for feeding on birds (Tempelis, 1975; Jansen et al., 2009b), whereas *Aedes* tend to be more associated with humans and are more commonly found in lower traps. Despite these factors contributing to variability, we did not observe differences in virome composition between ground and canopy traps. This finding suggests that, although mosquito feeding behaviors differ with respect to preferred feeding heights, their core virome composition tends to remain conserved across these environmental factors. This result highlights that, regardless of flight height preference, the virome may be primarily shaped by other factors such as mosquito species or environmental factors not directly tied to the specific collection height.

Nevertheless, we noted the presence of certain generalist viruses. We found three viruses concomitantly among the three genera. The *Culex Iflavi-like virus 4*, an *Iflavirus* identified previously in *Culex* species, we reported here for the first time in *Aedes* and *Anopheles* mosquitoes. Additionally, *Brevihamaparvovirus*, reported only once in a viral metagenomic study of wild bird (*Phylloscopus proregulus*) fecal samples, and although its host origin is still unknown, our data suggest that it can infect different mosquitoes’ species. Furthermore, the *Soybean thrips tombus-like virus 2*, identified only once in a study of the virome of the insect Thrips (Thekke-Veetil et al., 2020), could also potentially be adapted to infect other insect hosts, such as mosquitoes. Regarding the collection site and trap height, *Culex Iflavi-like virus 4*, *Hattula totivirus 2*, and *Mika virus* showed wide geographical distribution. *Hattula totivirus 2* was previously identified in *Ochlerotatus* mosquitoes in a study in Finland, while *Mika virus*, an unclassified virus of the *Totiviridae* family, was exclusively reported in the *Culex* genus in a study involving various mosquito species in Florida, USA (Batson et al., 2021). This corroborates the many virus species found here that were previously associated with hosts different from those collected in this study (**Figure 9**; **Supplementary Table S7**). Although most mosquito-associated viruses maintain an intrinsic ecological relationship with one or a few physiologically related hosts, resulting in a core virome within the host, several viruses have been observed with a broad host insect range. However, further studies are needed to confirm the host origin and host range of these viruses.

The identification and analysis of viromes in insects, including mosquitoes, can be significantly influenced by the presence of endogenous viral elements (EVEs). EVEs are remnants of viral genomes integrated into host DNA over evolutionary time. These elements can occasionally be transcribed, complicating the differentiation between truly circulating viruses and these genomic relics. For instance, EVEs derived from RNA viruses, such as those within the *Flaviviridae* and *Bunyaviridae* families, have been reported in mosquitoes and other insects (Katzourakis and Gifford, 2010; Wallau, 2022). Distinguishing EVEs from active viruses typically involves cross-referencing viral genome data with host genomic sequences, which has been demonstrated in studies analyzing small RNA datasets and host genome assemblies (Zakrzewski et al., 2018; da Silva et al., 2021). Despite these challenges, the identification of complete viral genomes or large contiguous fragments provides strong evidence for bona fide circulating viruses. This is because most EVEs are represented by short, fragmented sequences rather than complete viral genomes (Ter Horst et al., 2019). Thus, while some viral contigs identified in virome studies may require additional validation, the presence of longer sequences strengthens the case for the discovery of active viruses.

Our study has several limitations that should be acknowledged. First, there was a greater number of samples from the *Culex* genera compared to *Aedes* and *Anopheles*, which may have introduced bias in our findings. The limited number of pools analyzed presents a constraint in fully capturing the diversity and dynamics of mosquito viromes. While consistent patterns of host-specific virome composition were observed, a larger pool size in future studies would enhance the statistical power and allow for a more comprehensive understanding of site-specific and trap height influences. Additionally, we did not collect data on environmental parameters such as mosquito population size, temperature, and humidity, which could significantly influence viral presence and abundance. Lastly, we did not assess the presence of non-viral microorganisms that might affect virus-mosquito interactions, potentially overlooking important factors that could impact our results.

## 5 Conclusion

The composition of the mosquito virome was primarily influenced by the genus of the mosquito host (*Aedes*, *Anopheles*, and *Culex*), rather than by specific mosquito species, the collection site (within a 1-km radius), or the collection height (ground vs. canopy levels). However, the limited representation of *Aedes* and *Anopheles* pools may constrain the generalization of these findings across all mosquito genera. While the study’s geographically restricted setting (e.g., a zoo) appears to have a minimal effect on the mosquito-virus relationship despite environmental variability, it highlights key interactions between mosquitoes, their environment, and viromes. These findings contribute valuable insights into mosquito-virus dynamics and provide a foundation for future research and improved strategies to combat mosquito-borne diseases.

## Data Availability

The original contributions presented in the study are included in the article/**Supplementary Material**. Further inquiries can be directed to the corresponding authors.

## References

[B1] Adegbola R. O. Keith C. V. Gutierrez O. A. Goenaga R. Brown J. K. (2022). A previously undescribed polerovirus (Solemoviridae) infecting theobroma cacao germplasm. Plant Dis. 107. doi: 10.1094/PDIS-06-22-1449-PDN

[B2] Agboli E. Leggewie M. Altinli M. Schnettler E. (2019). Mosquito-specific viruses-transmission and interaction. Viruses 11, 873. doi: 10.3390/v11090873 31533367 PMC6784079

[B3] Alencar J. Mello C. F. Barbosa L. S. Gil-Santana H. R. Maia D. A. Marcondes C. B.. (2016). Diversity of yellow fever mosquito vectors in the Atlantic Forest of Rio de Janeiro, Brazil. Rev. Soc. Bras. Med. Trop 49, 351–356. doi: 10.1590/0037-8682-0438-2015 27384833

[B4] Almeida J. P. Aguiar E. R. Armache J. N. Olmo R. P. Marques J. T. (2021). The virome of vector mosquitoes. Curr. Opin. Virol 49, 7–12. doi: 10.1016/j.coviro.2021.04.002 33991759

[B5] Barcellos C. Matos V. Lana R. M. Lowe R. (2024). Climate change, thermal anomalies, and the recent progression of dengue in Brazil. Sci. Rep 14, 5948. doi: 10.1038/s41598-024-56044-y 38467690 PMC10928122

[B6] Batovska J. Blacket M. J. Brown K. Lynch S. E. (2016). Molecular identification of mosquitoes (Diptera: Culicidae) in southeastern Australia. Ecol. Evol 6, 3001–3011. doi: 10.1002/ece3.2095 27217948 PMC4863023

[B7] Batson J. Dudas G. Haas-Stapleton E. Kistler A. L. Li L. M. Logan P.. (2021). Single mosquito metatranscriptomics identifies vectors, emerging pathogens and reservoirs in one assay. Elife. 10, e68353. doi: 10.7554/eLife.68353 33904402 PMC8110308

[B8] Bethesda M. D. (2004). NCBI virus (National Center for Biotechnology Information: National Library of Medicine (US). Available online at: https://www.ncbi.nlm.nih.gov/labs/virus/vssi// (Accessed March 15, 2024).

[B9] Bolger A. M. Lohse M. Usadel B. (2014). Trimmomatic: a flexible trimmer for Illumina sequence data. Bioinformatics 30, 2114–2120. doi: 10.1093/bioinformatics/btu170 24695404 PMC4103590

[B10] Bolling B. G. Olea-Popelka F. J. Eisen L. Moore C. G. Blair C. D. (2012). Transmission dynamics of an insect-specific flavivirus in a naturally infected Culex pipiens laboratory colony and effects of co-infection on vector competence for West Nile virus. Virology 427, 90–97. doi: 10.1016/j.virol.2012.02.016 22425062 PMC3329802

[B11] Brady O. J. Hay S. I. (2020). The global expansion of dengue: how aedes aEgypti mosquitoes enabled the first pandemic arbovirus. Annu. Rev. Entomol 65, 191–208. doi: 10.1146/annurev-ento-011019-024918 31594415

[B12] Brasil. Ministério da Saúde (2023). Boletim epidemiológico. Available online at: https://www.gov.br/saude/pt-br/centrais-de-conteudo/publicacoes/boletins/epidemiologicos/edicoes/2023/boletim-epidemiologico-volume-54-no-13/ (Accessed June 10, 2024).

[B13] Brasil. Ministério da Saúde (2024). Informe Semanal n° 14. Available online at: https://www.gov.br/saude/pt-br/assuntos/saude-de-a-a-z/a/arboviroses/informe-semanal/informe-semanal-no-14.pdf/view (Accessed June 10, 2024).

[B14] Buchfink B. Xie C. Huson D. H. (2015). Fast and sensitive protein alignment using DIAMOND. Nat. Methods 12, 59–60. doi: 10.1038/nmeth.3176 25402007

[B15] Bushmanova E. Antipov D. Lapidus A. Prjibelski A. D. (2019). rnaSPAdes: a *de novo* transcriptome assembler and its application to RNA-Seq data. Gigascience 8, giz100. doi: 10.1093/gigascience/giz100 31494669 PMC6736328

[B16] Conn J. E. Wilkerson R. C. Segura M. N. de Souza R. T. Schlichting C. D. Wirtz R. A.. (2002). Emergence of a new neotropical malaria vector facilitated by human migration and changes in land use. Am. J. Trop. Med. Hyg 66, 18–22. doi: 10.4269/ajtmh.2002.66.18 12135261

[B17] Consoli R. A. G. B. Lourenço-de-Oliveira R. (1994). Principais Mosquitos de Importância Sanitária No Brasil; Cadernos de Saúde Pública (Rio de Janeiro, Brazil: FIOCRUZ).

[B18] Cook S. Chung B. Y. Bass D. Moureau G. Tang S. McAlister E. (2013). Novel virus discovery and genome reconstruction from field RNA samples reveals highly divergent viruses in dipteran hosts. PloS One 8, e80720. doi: 10.1371/journal.pone.0080720 24260463 PMC3832450

[B19] Cuesta E. B. Coulibaly B. Bukhari T. Eiglmeier K. Kone R. Coulibaly M. B.. (2021). Comprehensive ecological and geographic characterization of eukaryotic and prokaryotic microbiomes in african anopheles. Front. Microbiol 12. doi: 10.3389/fmicb.2021.635772 PMC8153677 34054746

[B20] Cui S. Liu Y. Zhao J. Peng X. Lu G. Shi W. (2022). An updated review on SARS-coV-2 infection in animals. Viruses 14, 1527. doi: 10.3390/v14071527 35891507 PMC9323600

[B21] da Silva A. F. Dezordi F. Z. MaChado L. C. de Oliveira R. D. Qin S. Fan H.. (2021). Metatranscriptomic analysis identifies different viral-like sequences in two neotropical Mansoniini mosquito species. Virus Res 301, 198455. doi: 10.1016/j.virusres.2021.198455 34015364

[B22] de Oliveira-Ferreira J. Lourenço-de-Oliveira R. Teva A. Deane L. M. Daniel-Ribeiro C. T. (1990). Natural malaria infections in anophelines in Rondonia State, Brazilian Amazon. Am. J. Trop. Med. Hyg 43, 6–10. doi: 10.4269/ajtmh.1990.43.1.TM0430010006 2200290

[B23] Duarte A. M. Pereira D. M. de Paula M. B. Fernandes A. Urbinatti P. R. Ribeiro A. F.. (2013). Natural infection in anopheline species and its implications for autochthonous malaria in the Atlantic Forest in Brazil. Parasit Vectors 6, 58. doi: 10.1186/1756-3305-6-58 23497493 PMC3605261

[B24] Dudas G. Obbard D. J. (2015). Are arthropods at the heart of virus evolution? Elife 4, e06837. doi: 10.7554/eLife.06837 25751043 PMC4352705

[B25] Feng Y. Gou Q. Y. Yang W. H. Wu W. C. Wang J. Holmes E. C.. (2022). A time-series meta-transcriptomic analysis reveals the seasonal, host, and gender structure of mosquito viromes. Virus Evol 8, veac006. doi: 10.1093/ve/veac006 35242359 PMC8887699

[B26] Forattini O. P. (2002). Culicidologia médica Vol. 2 (São Paulo: EdUSP), 860.

[B27] Fort P. Albertini A. Van-Hua A. Berthomieu A. Roche S. Delsuc F.. (2012). Fossil rhabdoviral sequences integrated into arthropod genomes: ontogeny, evolution, and potential functionality. Mol. Biol. Evol 29, 381–390. doi: 10.1093/molbev/msr226 21917725

[B28] Gandrud C. (2020). Reproducible research with R and RStudio (Chapman & Hall/CRC the R series) 3rd edition. ISBN 0367144026, 9780367144029. doi: 10.1201/9780429031854

[B29] Gao C.-H. Chen C. Akyol T. Dusa A. Yu G. Cao B.. (2024). ggVennDiagram: Intuitive Venn diagram software extended. iMeta 3, e177. doi: 10.1002/imt2.177 38868514 PMC10989133

[B30] Ghurye J. S. Cepeda-Espinoza V. Pop M. (2016). Metagenomic assembly: overview, challenges and applications. Yale J. Biol. Med 89, 353–362. 27698619 PMC5045144

[B31] Greenwood A. D. Tsangaras K. Ho S. Y. Szentiks C. A. Nikolin V. M. Ma G.. (2012). A potentially fatal mix of herpes in zoos. Curr. Biol 22, 1727–1731. doi: 10.1016/j.cub.2012.07.035 22902751

[B32] Guimarães L. O. Simões R. F. Chagas C. R. F. Menezes R. M. T. Silva F. S. Monteiro E. F.. (2021). Assessing diversity, plasmodium infection and blood meal sources in mosquitoes (Diptera: culicidae) from a Brazilian zoological park with avian malaria transmission. Insects 12, 215. doi: 10.3390/insects12030215 33802320 PMC7999885

[B33] Guo M. Yuan X. Song Y. Liu Y. Wang X. F. (2022). First report of maize yellow mosaic virus (MaYMV) naturally infecting wheat in China. Plant Dis. 106. doi: 10.1094/PDIS-12-21-2774-PDN

[B34] Hall-Mendelin S. McLean B. J. Bielefeldt-Ohmann H. Hobson-Peters J. Hall R. A. van den Hurk A. F. (2016). The insect-specific Palm Creek virus modulates West Nile virus infection in and transmission by Australian mosquitoes. Parasit Vectors 9, 414. doi: 10.1186/s13071-016-1683-2 27457250 PMC4960669

[B35] Hameed M. Liu K. Anwar M. N. Wahaab A. Li C. Di D.. (2020). A viral metagenomic analysis reveals rich viral abundance and diversity in mosquitoes from pig farms. Transbound Emerg. Dis 67, 328–343. doi: 10.1111/tbed.13355 31512812

[B36] Harbach R. E. (2013). Mosquito taxonomic inventory. Available online at: https://mosquito-taxonomic-inventory.myspecies.info/ (Accessed 19 January 2024).

[B37] Hobson-Peters J. Yam A. W. Lu J. W. Setoh Y. X. May F. J. Kurucz N.. (2013). A new insect-specific flavivirus from northern Australia suppresses replication of West Nile virus and Murray Valley encephalitis virus in co-infected mosquito cells. PloS One 8, e56534. doi: 10.1371/journal.pone.0056534 23460804 PMC3584062

[B38] Ito K. Murphy D. (2013). Application of ggplot2 to pharmacometric graphics. CPT Pharmacometrics Syst. Pharmacol 2, e79. doi: 10.1038/psp.2013.56 24132163 PMC3817376

[B39] Jansen C. C. Webb C. E. Graham G. C. Craig S. B. Zborowski P. Ritchie S. A.. (2009b). Blood sources of mosquitoes collected from urban and peri-urban environments in eastern Australia with species-specific molecular analysis of avian blood meals. Am. J. Trop. Med. Hyg 81, 849–857. doi: 10.4269/ajtmh.2009.09-0008 19861621

[B40] Jansen C. C. Zborowski P. Ritchie S. A. Van Den Hurk A. F. (2009). Efficacy of bird-baited traps placed at different heights for collecting ornithophilic mosquitoes in eastern Queensland, Australia. Aust. J. Entomology 48, 53–59. doi: 10.1111/j.1440-6055.2008.00671.x

[B41] Johnson K. N. Johnson K. L. Dasgupta R. Gratsch T. Ball L. A. (2001). Comparisons among the larger genome segments of six nodaviruses and their encoded RNA replicases. J. Gen. Virol 82, 1855–1866. doi: 10.1099/0022-1317-82-8-1855 11457991

[B42] Katzourakis A. Gifford R. J. (2010). Endogenous viral elements in animal genomes. PloS Genet 6, e1001191. doi: 10.1371/journal.pgen.1001191 21124940 PMC2987831

[B43] Kolde R. (2019). “Pheatmap: Pretty heatmaps,” in R package version 1.0.12. Available at: https://CRAN.R-project.org/package=pheatmap (Accessed January 19, 2024).

[B44] Kurth A. Wibbelt G. Gerber H. P. Petschaelis A. Pauli G. Nitsche A. (2008). Rat-to-elephant-to-human transmission of cowpox virus. Emerg. Infect. Dis 14, 670–671. doi: 10.3201/eid1404.070817 18394293 PMC2570944

[B45] Kuwata R. Isawa H. Hoshino K. Sasaki T. Kobayashi M. Maeda K.. (2015). Analysis of mosquito-borne flavivirus superinfection in culex tritaeniorhynchus (Diptera: culicidae) cells persistently infected with culex flavivirus (Flaviviridae). J. Med. Entomol 52, 222–229. doi: 10.1093/jme/tju059 26336307

[B46] Lane J. (1953). Neotropical culicidae (São Paulo: Universidade de São Paulo, 2v).

[B47] Lee H. I. Seo B. Y. Burkett D. A. Lee W. J. Shin Y. H. (2006). Study of flying height of culicid species in the northern part of the Republic of Korea. J. Am. Mosq. Control Assoc 22, 239–245. doi: 10.2987/8756-971X(2006)22[239:SOFHOC]2.0.CO;2 17019769

[B48] Lequime S. Lambrechts L. (2017). Discovery of flavivirus-derived endogenous viral elements in Anopheles mosquito genomes supports the existence of Anopheles-associated insect-specific flaviviruses. Virus Evol 3, vew035. doi: 10.1093/ve/vew035 28078104 PMC5217911

[B49] Li C. X. Shi M. Tian J. H. Lin X. D. Kang Y. J. Chen L. J.. (2015). Unprecedented genomic diversity of RNA viruses in arthropods reveals the ancestry of negative-sense RNA viruses. Elife. 4, e05378. doi: 10.7554/eLife.05378 25633976 PMC4384744

[B50] Marceló-Díaz C. Morales C. A. Lesmes M. C. Fuya P. Mendez S. A. Cadena H.. (2022). Arbovirus vectors in municipalities with a high risk of dengue in Cauca, Southwestern Colombia. GigaByte 2022, gigabyte53. doi: 10.46471/gigabyte.53 36824502 PMC9930565

[B51] Marklewitz M. Zirkel F. Kurth A. Drosten C. Junglen S. (2015). Evolutionary and phenotypic analysis of live virus isolates suggests arthropod origin of a pathogenic RNA virus family. Proc. Natl. Acad. Sci. U S A 2112, 7536–7541. doi: 10.1073/pnas.1502036112 PMC4475995 26038576

[B52] McMurdie P. J. Holmes S. (2013). phyloseq: an R package for reproducible interactive analysis and graphics of microbiome census data. PloS One 8, e61217. doi: 10.1371/journal.pone.0061217 23630581 PMC3632530

[B53] Mirza J. D. Guimarães L. O. Wilkinson S. Rocha E. C. Bertanhe M. Helfstein V. C.. (2024). Tracking arboviruses, their transmission vectors and potential hosts by nanopore sequencing of mosquitoes. Microb. Genom 10, 1184. doi: 10.1099/mgen.0.001184 PMC10868619 38240642

[B54] Moore T. C. Brown H. E. (2022). Estimating aedes aEgypti (Diptera: culicidae) flight distance: meta-data analysis. J. Med. Entomol 59, 1164–1170. doi: 10.1093/jme/tjac070 35640992

[B55] Nasar F. Erasmus J. H. Haddow A. D. Tesh R. B. Weaver S. C. (2015). Eilat virus induces both homologous and heterologous interference. Virology 484, 51–58. doi: 10.1016/j.virol.2015.05.009 26068885 PMC4567418

[B56] Nga P. T. Parquet Mdel C. Lauber C. Parida M. Nabeshima T. Yu F.. (2011). Discovery of the first insect nidovirus, a missing evolutionary link in the emergence of the largest RNA virus genomes. PloS Pathog 7, e1002215. doi: 10.1371/journal.ppat.1002215 21931546 PMC3169540

[B57] Nolting J. M. Dennis P. Long L. Holtvoigt L. Brown D. King M. J.. (2013). Low pathogenic influenza A virus activity at avian interfaces in Ohio zoos 2006-2009. Avian Dis 57, 657–662. doi: 10.1637/10528-031313-Reg.1 24283133

[B58] Nurk S. Meleshko D. Korobeynikov A. Pevzner P. A. (2017). metaSPAdes: a new versatile metagenomic assembler. Genome Res 27, 824–834. doi: 10.1101/gr.213959.116 28298430 PMC5411777

[B59] Oguzie J. U. Nwangwu U. C. Oluniyi P. E. Olumade T. J. George U. E. Kazeem A.. (2022). Metagenomic sequencing characterizes a wide diversity of viruses in field mosquito samples in Nigeria. Sci. Rep 12, 7616. doi: 10.1038/s41598-022-11797-2 35538241 PMC9090917

[B60] Öhlund P. Lundén H. Blomström A. L. (2019). Insect-specific virus evolution and potential effects on vector competence. Virus Genes 55, 127–137. doi: 10.1007/s11262-018-01629-9 30632016 PMC6458977

[B61] Oksanen J. Kindt R. Legendre P. O'Hara B. Simpson G. L. Solymos P.. (2008). Vegan: community ecology package, R package version 1. Available online at: http://vegan.r-forge.r-project.org/ http://cran.r-project.org/.

[B62] Paulson J. N. Stine O. C. Bravo H. C. Pop M. (2013). Differential abundance analysis for microbial marker-gene surveys. Nat. Methods 10, 1200–1202. doi: 10.1038/nmeth.2658 24076764 PMC4010126

[B63] Pettersson J. H. Shi M. Eden J. S. Holmes E. C. Hesson J. C. (2019). Meta-Transcriptomic Comparison of the RNA Viromes of the Mosquito Vectors Culex pipiens and Culex torrentium in Northern Europe. Viruses 11, 1033. doi: 10.3390/v11111033 31698792 PMC6893722

[B64] Ribeiro G. O. Morais V. S. Monteiro F. J. C. Ribeiro E. S. D. Rego M. O. D. S. Souto R. N. P.. (2020). Aedes aEgypti from amazon basin harbor high diversity of novel viral species. Viruses 12, 866. doi: 10.3390/v12080866 32784421 PMC7472207

[B65] Rocklöv J. Dubrow R. (2020). Climate change: an enduring challenge for vector-borne disease prevention and control. Nat. Immunol 21, 479–483. doi: 10.1038/s41590-020-0648-y 32313242 PMC7223823

[B66] Root F. M. (1926). Studies on Brazilian mosquitoes. I. The anophelines of the nyssorhynchus group. Am. J. Epidemiol 6, 684–717. doi: 10.1093/oxfordjournals.aje.a120038

[B67] Sadeghi M. Altan E. Deng X. Barker C. M. Fang Y. Coffey L. L.. (2018). Virome of > 12 thousand Culex mosquitoes from throughout California. Virology 523, 74–88. doi: 10.1016/j.virol.2018.07.029 30098450

[B68] Sahul Hameed A. S. Ninawe A. S. Nakai T. Chi S. C. Johnson K. L. (2019). Ictv report consortium. ICTV Virus Taxonomy Profile: Nodaviridae. J. Gen. Virol 100, 3–4. doi: 10.1099/jgv.0.001170 30431412 PMC12662032

[B69] Schultz M. J. Frydman H. M. Connor J. H. (2018). Dual Insect specific virus infection limits Arbovirus replication in Aedes mosquito cells. Virology 518, 406–413. doi: 10.1016/j.virol.2018.03.022 29625404 PMC8005325

[B70] Shi C. Beller L. Deboutte W. Yinda K. C. Delang L. Vega-Rúa A.. (2019). Stable distinct core eukaryotic viromes in different mosquito species from Guadeloupe, using single mosquito viral metagenomics. Microbiome 7, 121. doi: 10.1186/s40168-019-0734-2 31462331 PMC6714450

[B71] Shi M. Lin X. D. Tian J. H. Chen L. J. Chen X. Li C. X.. (2016). Redefining the invertebrate RNA virosphere. Nature 540, 539–543. doi: 10.1038/nature20167 27880757

[B72] Shi C. Liu Y. Hu X. Xiong J. Zhang B. Yuan Z. (2015). A metagenomic survey of viral abundance and diversity in mosquitoes from Hubei province. PloS One 10, e0129845. doi: 10.1371/journal.pone.0129845 26030271 PMC4452694

[B73] Shi C. Zhao L. Atoni E. Zeng W. Hu X. Matthijnssens J.. (2020). Stability of the Virome in Lab- and Field-Collected Aedes albopictus Mosquitoes across Different Developmental Stages and Possible Core Viruses in the Publicly Available Virome Data of Aedes Mosquitoes. mSystems 5, e00640–e00620. doi: 10.1128/mSystems.00640-20 32994288 PMC7527137

[B74] Smith J. L. Fonseca D. M. (2004). Rapid assays for identification of members of the Culex (Culex) pipiens complex, their hybrids, and other sibling species (Diptera: culicidae). Am. J. Trop. Med. Hyg 70, 339–345. doi: 10.4269/ajtmh.2004.70.339 15100444

[B75] Snow W. F. (1977). [amp]]lsquo;The height and direction of flight of mosquitoes in West African savanna, in relation to wind speed and direction’. Bull. Entomological Res 67, 271–279. doi: 10.1017/S0007485300011081

[B76] Snow W. F. (1979). The vertical distribution of flying mosquitoes (Diptera: Culicidae) near an area of irrigated rice-fields in the Gambia. Bull. Entomological Res 69, 561–571. doi: 10.1017/S0007485300020113

[B77] Sõmera M. Fargette D. Hébrard E. Sarmiento C. (2021). Ictv report consortium. ICTV virus taxonomy profile: solemoviridae 2021. J. Gen. Virol 102, 1707. doi: 10.1099/jgv.0.001707 PMC8744267 34951396

[B78] Souza-Neto J. A. Powell J. R. Bonizzoni M. (2019). Aedes aEgypti vector competence studies: A review. Infect. Genet. Evol 67, 191–209. doi: 10.1016/j.meegid.2018.11.009 30465912 PMC8135908

[B79] Swanson D. A. Adler P. H. (2010). Vertical distribution of haematophagous Diptera in temperate forests of the southeastern U.S.A. Med. Vet. Entomol 24, 182–188. doi: 10.1111/j.1365-2915.2010.00862.x 20374479

[B80] Tempelis C. H. (1975). Host-feeding patterns of mosquitoes, with a review of advances in analysis of blood meals by serology. J. Med. Entomol 11, 635–653. doi: 10.1093/jmedent/11.6.635 235647

[B81] Ter Horst A. M. Nigg J. C. Dekker F. M. Falk B. W. (2019). Endogenous viral elements are widespread in arthropod genomes and commonly give rise to PIWI-interacting RNAs. J. Virol 93, e02124–e02118. doi: 10.1128/JVI.02124-18 30567990 PMC6401445

[B82] Thekke-Veetil T. Lagos-Kutz D. McCoppin N. K. Hartman G. L. Ju H.-K. Lim H.-S.. (2020). Soybean thrips (Thysanoptera: thripidae) harbor highly diverse populations of arthropod, fungal and plant viruses. Viruses 12, 1376. doi: 10.3390/v12121376 33271916 PMC7761488

[B83] Thomas J. E. Gronenborn B. Harding R. M. Mandal B. Grigoras I. Randles J. W.. (2021). Ictv report consortium. ICTV Virus Taxonomy Profile: Nanoviridae. J. Gen. Virol 102, 1544. doi: 10.1099/jgv.0.001544 PMC8515864 33433311

[B84] Thongsripong P. Chandler J. A. Kittayapong P. Wilcox B. A. Kapan D. D. Bennett S. N. (2021). Metagenomic shotgun sequencing reveals host species as an important driver of virome composition in mosquitoes. Sci. Rep 11, 8448. doi: 10.1038/s41598-021-87122-0 33875673 PMC8055903

[B85] Tuno N. Githeko A. K. Nakayama T. Minakawa N. Takagi M. Yan G. (2006). The association between the phytoplankton, Rhopalosolen species (Chlorophyta; Chlorophyceae), and Anopheles Gambiae sensu lato (Diptera: Culicidae) larval abundance in western Kenya. Ecol. Res 21, 476–482. doi: 10.1007/s11284-005-0131-0

[B86] Urakova N. Brustolin M. Joseph R. E. Johnson R. M. Pujhari S. Rasgon J. L. (2020). Anopheles Gambiae densovirus (AgDNV) negatively affects Mayaro virus infection in Anopheles Gambiae cells and mosquitoes. Parasit Vectors 13, 210. doi: 10.1186/s13071-020-04072-8 32321560 PMC7178629

[B87] Valles S. M. Chen Y. Firth A. E. Guérin D. M. A. Hashimoto Y. Herrero S.. (2017). Ictv report consortium. ICTV Virus Taxonomy Profile: Iflaviridae. J. Gen. Virol 98, 527–528. doi: 10.1099/jgv.0.000757 28382900 PMC5657024

[B88] Wallau G. L. (2022). RNA virus EVEs in insect genomes. Curr. Opin. Insect Sci 49, 42–47. doi: 10.1016/j.cois.2021.11.005 34839033

[B89] World Health Organization (2020). Vector-borne diseases. Available online at: http://www.who.int/mediacentre/factsheets/fs387/en/ (Accessed June 10, 2024).

[B90] Wu H. Pang R. Cheng T. Xue L. Zeng H. Lei T.. (2020). Abundant and diverse RNA viruses in insects revealed by RNA-seq analysis: ecological and evolutionary implications. mSystems 5, e00039–e00020. doi: 10.1128/mSystems.00039-20 32636338 PMC7343303

[B91] Yang X. Qin S. Liu X. Zhang N. Chen J. Jin M.. (2023). Meta-viromic sequencing reveals virome characteristics of mosquitoes and culicoides on zhoushan island, China. Microbiol. Spectr 11, e0268822. doi: 10.1128/spectrum.02688-22 36651764 PMC9927462

[B92] Zakrzewski M. Rašić G. Darbro J. Krause L. Poo Y. S. Filipović I.. (2018). Mapping the virome in wild-caught Aedes aEgypti from Cairns and Bangkok. Sci. Rep 8, 4690. doi: 10.1038/s41598-018-22945-y 29549363 PMC5856816

