# Supplementary material for: Exploring mosquito virome dynamics within São Paulo Zoo: insights into mosquito-virus-environment interactions
Source: Front Cell Infect Microbiol. 2025 Jan 10;14:1496126. doi: 10.3389/fcimb.2024.1496126 (PMC11757883; doi:10.3389/fcimb.2024.1496126)
Supplement: Supplementary file 9 [file Table9.docx]

**Supplemental Figures:**

**Exploring Mosquito Virome Dynamics within São Paulo Zoo: Insights into Mosquito-Virus-Environment Interactions**

Lilian de Oliveira Guimarães^†,1*^, Geovani de Oliveira Ribeiro^†,2,3^, Roseane da Couto^4^, Endrya do Socorro Foro Ramos^4^, Vanessa dos Santos Morais^5^, Juliana Telles-de-Deus^1^, Vanessa Christe Helfstein^1^, Jesus Maia dos Santos^1^, Xutao Deng^6,7^, Eric Delwart^7^, Ramendra Pati Pandey^8^, Vera Lucia Fonseca de Camargo-Neves^1^, Antonio Charlys da Costa^‡,5^, Karin Kirchgatter^‡,1,5^, Élcio Leal^‡,4^

^1^Instituto Pasteur, São Paulo, SP 01027-000, Brazil

^2^General-Coordination of Public Health Laboratories, Health and Environment Surveillance Secretariat, Ministry of Health, Brasilia, Distrito Federal, Brazil;

^3^Department of Cellular Biology, University of Brasilia (UNB), Brasilia, Distrito Federal, Brazil;

^4^Institute of Biological Sciences, Federal University of Pará, Belem, Pará, Brazil.

^5^Instituto de Medicina Tropical, Faculdade de Medicina, Universidade de São Paulo, São Paulo, SP 05403-000, Brazil

^6^Vitalant Research Institute, San Francisco, CA 94143, USA

^7^Department Laboratory Medicine, University of California San Francisco, San Francisco, CA 94143, USA

^8^School of Health Sciences and Technology (SoHST), University of Petroleum and Energy Studies, UPES, Bidholi, Dehradun 248007, Uttarakhand, India.

^†^These authors contributed equally to this work.

^‡^These authors jointly supervised this work.

*Author to whom correspondence should be addressed:

Karin Kirchgatter. Instituto Pasteur, Rua Paula Sousa 166, São Paulo 01027-000, Brazil. karink@usp.br

**
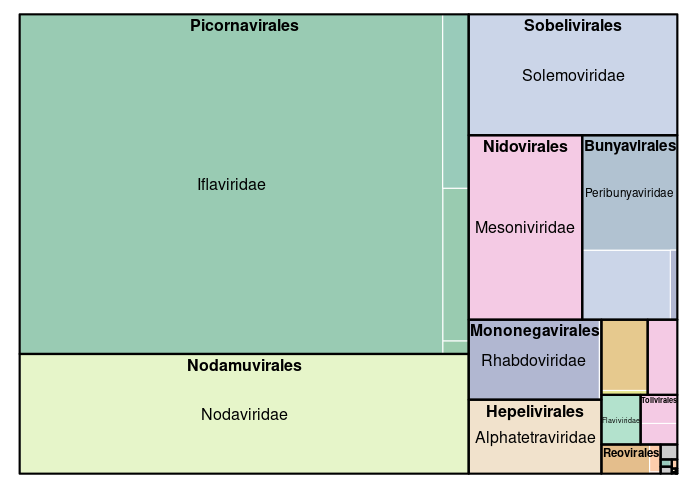
**

**Figure S1**: Treemap plot of proportion reads recovered among viral order (bold) and family from the 26 mosquitoes pools.


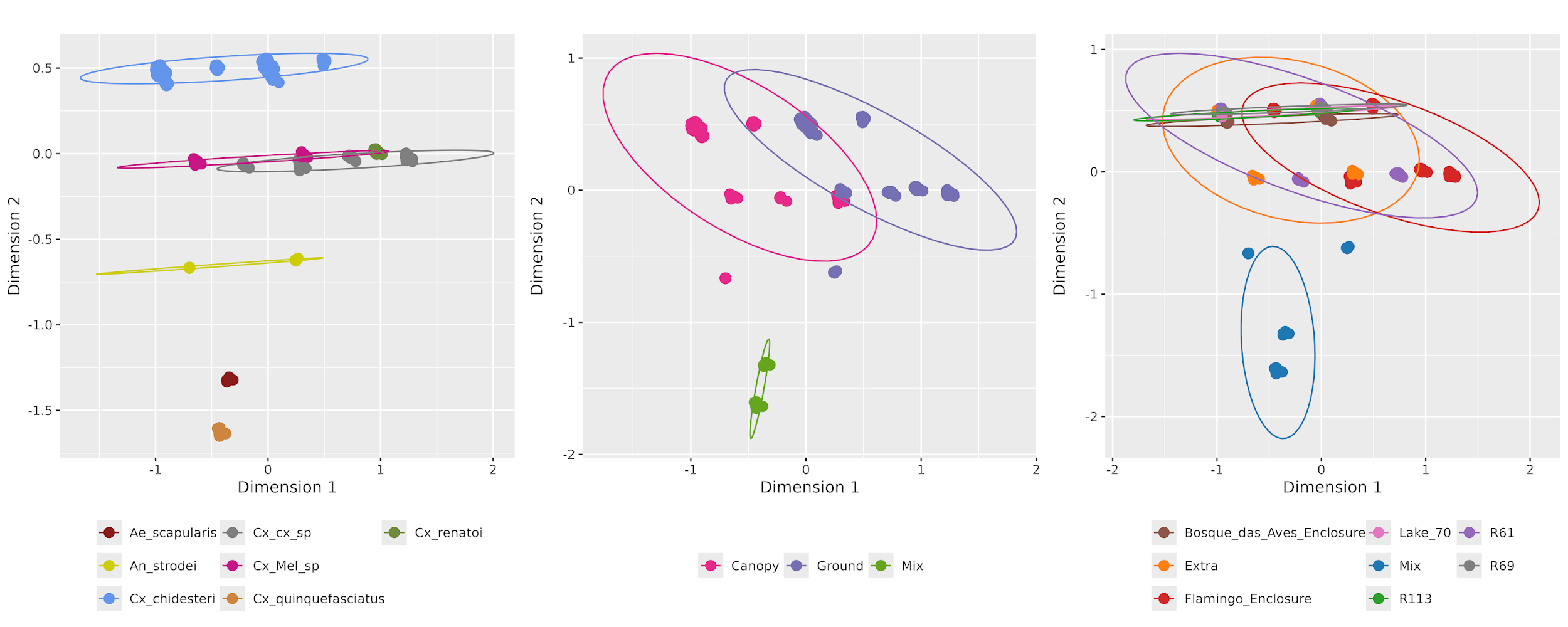


**Figure S2:** Non-metric multidimensional scaling (NMDS) for viral composition on viral species level by host species. Circles show 95% normal probability ellipse for each species group (bottom panel).

**
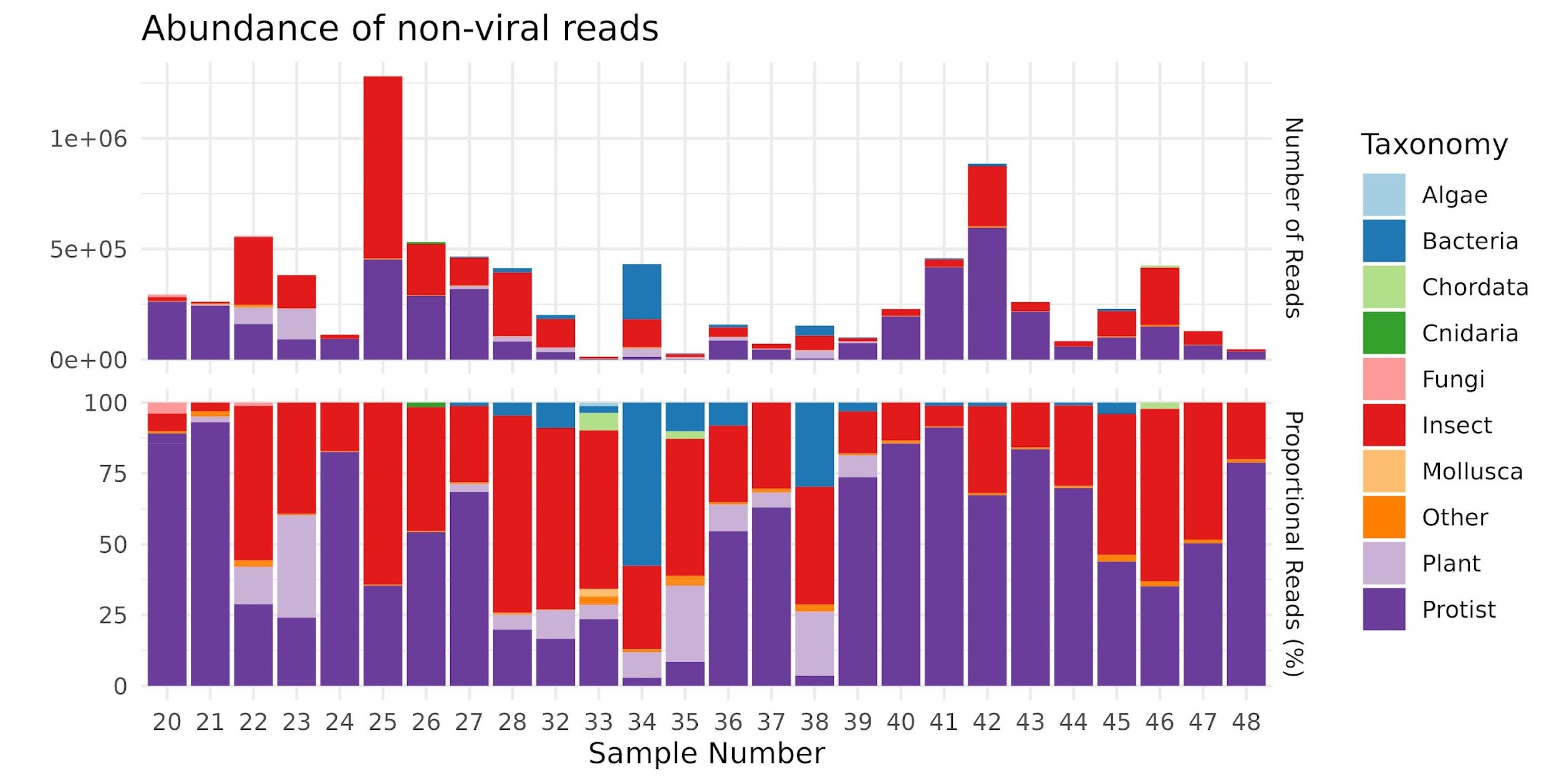
**

**Figure S3**: Absolute (above) and relative abundance (below) of non-viral reads from twenty-six mosquitoes pools. The abundance of non-viral groups was estimated computing the number each group divides by total non-viral reads from each pool.


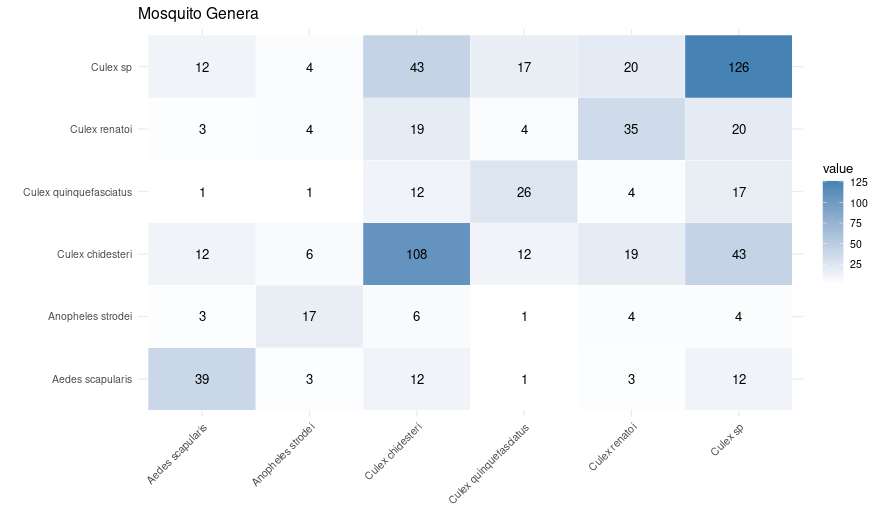


**Figure S4**: Number of viral coinfections in mosquitoes. The heatmap shows the total number of viruses. Numbers within each rectangle are the total number of viruses found different mosquitoes. Rectangle in the diagonal represent the number of viruses found in a single species.
